# Effect of ring rotation upon gas adsorption in SIFSIX-3-M (M = Fe, Ni) pillared square grid networks†
†Electronic supplementary information (ESI) available. CCDC 1517363–1517366. For ESI and crystallographic data in CIF or other electronic format see DOI: 10.1039/c6sc05012c



**DOI:** 10.1039/c6sc05012c

**Published:** 2016-12-19

**Authors:** Sameh K. Elsaidi, Mona H. Mohamed, Cory M. Simon, Efrem Braun, Tony Pham, Katherine A. Forrest, Wenqian Xu, Debasis Banerjee, Brian Space, Michael J. Zaworotko, Praveen K. Thallapally

**Affiliations:** a Chemistry Department , Faculty of Science , Alexandria University , P. O. Box 426 Ibrahimia , Alexandria 21321 , Egypt; b Physical and Computational Science Directorate , Pacific Northwest National Laboratory , Richland , WA 99352 , USA . Email: praveen.thallapally@pnnl.gov; c Department of Chemical and Biomolecular Engineering , University of California-Berkeley , Berkeley , CA 94720 , USA; d Department of Chemistry , CHE205 , University of South Florida , 4202 E. Fowler Avenue , Tampa , FL 33620 , USA; e X-ray Science Division , Advanced Photon Source , Argonne National Laboratory , Argonne , Illinois 60439 , USA; f Department of Chemical & Environmental Sciences , University of Limerick , Limerick , Republic of Ireland . Email: Michael.Zaworotko@ul.ie

## Abstract

Dynamic and flexible metal–organic frameworks (MOFs) that respond to external stimuli, such as stress, light, heat, and the presence of guest molecules, hold promise for applications in chemical sensing, drug delivery, gas separations, and catalysis. A greater understanding of the relationship between flexible constituents in MOFs and gas adsorption may enable the rational design of MOFs with dynamic moieties and stimuli-responsive behavior. Here, we detail the effect of subtle structural changes upon the gas sorption behavior of two “SIFSIX” pillared square grid frameworks, namely SIFSIX-3-M (M = Ni, Fe). We observe a pronounced inflection in the Xe adsorption isotherm in the Ni variant. With evidence from X-ray diffraction studies, density functional theory, and molecular simulations, we attribute the inflection to a disordered to ordered transition of the rotational configurations of the pyrazine rings induced by sorbate–sorbent interactions. We also address the effect of cage size, temperature, and sorbate on the guest-induced ring rotation and the adsorption isotherms. The absence of an inflection in the Xe adsorption isotherm in SIFSIX-3-Fe and in the Kr, N_2_, and CO_2_ adsorption isotherms in SIFSIX-3-Ni suggest that the inflection is highly sensitive to the match between the size of the cage and the guest molecule.

## Abstract 2

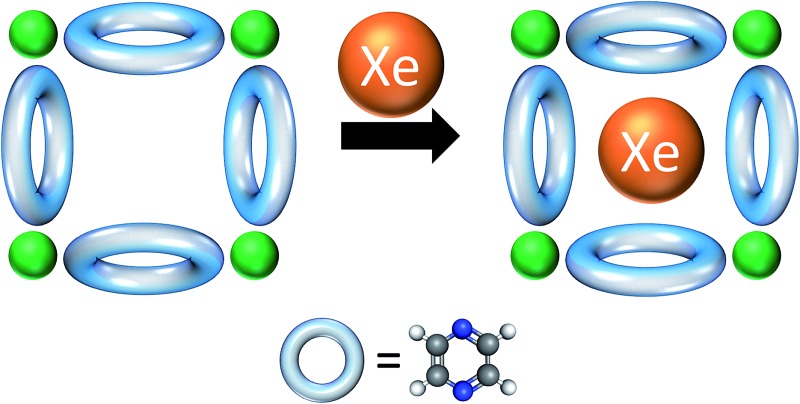
Dynamic and flexible metal–organic frameworks (MOFs) that respond to external stimuli, such as stress, light, heat, and the presence of guest molecules, hold promise for applications in chemical sensing, drug delivery, gas separations, and catalysis.

## Introduction

Metal–organic frameworks (MOFs) are composed of rigid organic linkers serving as struts and metal ions/metal clusters as nodes. A number of MOFs are dynamic and flexible in nature and respond to external stimuli such as mechanical stress, heat, gas ad-/de-sorption, and light.^1–8^ The structure and composition of MOFs influences their response, or lack thereof, to external stimuli.^9–14^ Understanding these relationships could enable us to engineer such dynamic properties into MOFs and exploit them for applications such as gas storage, chemical sensing, drug delivery, and luminescence.^15–22^ The dynamics of the organic linker can play a significant role in the properties of the MOF, including the adsorption and diffusion behavior, capacity, and selectivity.

Inflections, steps, and hysteresis have been reported in gas adsorption isotherms in MOFs as a signature of structural transitions upon gas adsorption. Such behavior has been attributed to gate opening and/or a breathing effect, where the MOF backbone flexes.^23–25^ Nevertheless, there are very few reports that have addressed the mechanism of the dynamics of organic linkers in the porous framework and how this can lead to non-Langmuirian adsorption isotherms.^26–30^ The dynamics of the organic linker during adsorption and desorption are influenced by sorbate–sorbent interactions and will therefore be sorbate dependent. Linker rotation is of relevance because it could potentially enhance the selective adsorption of certain guests;^26,31^ even modest swiveling of struts affects the pore size and geometry. Herein, we present experimental observations and computational studies of the influence of rotating pyrazine rings in the pillared square grid platform, SIFSIX-3-M, of formula [M(pyz)_2_SiF_6_] (M = Fe or Ni; pyz = pyrazine) during the adsorption of various gases such as Xe, Kr, CO_2_ and N_2_. These pillared MOFs have been widely investigated because their hybrid and ultramicroporous nature enable benchmark selectivity towards important industrial gases such as carbon dioxide, xenon and acetylene.^32–37^ The structural changes in such MOFs during adsorption of different gases remain largely unstudied and are addressed herein.

**Fig. 1:**
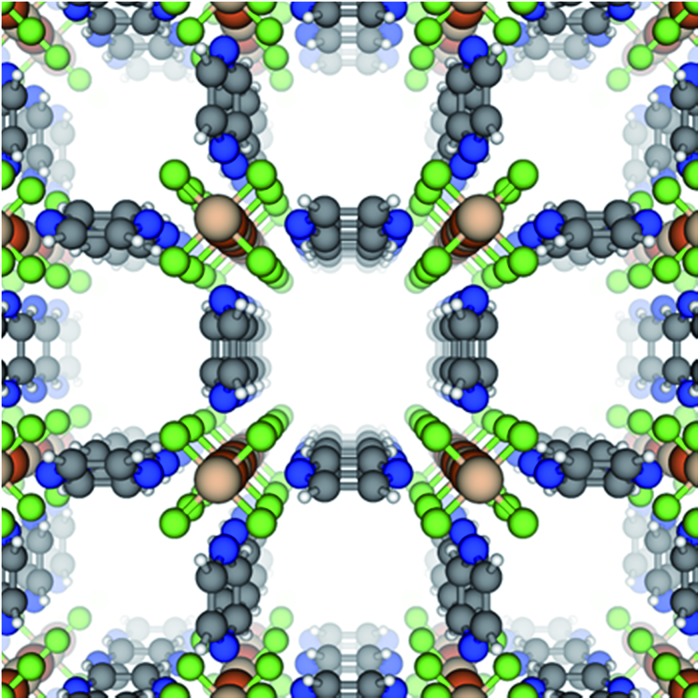
The crystal structure of SIFSIX-3-Fe viewed down the *c*-axis, which we define by the metal–SiF_6_ chain. Colors = {blue: N, gray: C, white: H, green: F, orange: S, tan: Fe}.

A new isostructure of the SIFSIX-3-M family,^32,33,38,39^ SIFSIX-3-Fe, [Fe(pyz)_2_(SiF_6_)] (Fig. 1), is synthesized, and its sorption behavior is compared with its Ni analogue, SIFSIX-3-Ni. SIFSIX-3-M networks are formed by M(pyz)_2_^2+^ type square grids, connected by SiF_6_^2–^ anions to form frameworks of primitive cubic, pcu, topology with pore diameters of 3.5–3.8 Å. Computational studies indicate that the best materials for Xe capture and Xe/Kr separation would exhibit pore sizes of ∼4 Å, close to the kinetic diameter of Xe.^40,41^ SIFSIX-3-Fe and SIFSIX-3-Ni were therefore evaluated with respect to Xe/Kr adsorption and separation.^42–47^

## Experimental

Full synthetic and characterization details are provided in the ESI.† All reagents were used as purchased. Solvents were purified according to standard methods and stored in the presence of molecular sieves. Gas adsorption was measured on Quantachrome Autosorb-1 volumetric gas adsorption analyzer.

### Synthesis of [Fe(pyz)_2_(SiF_6_)], SIFSIX-3-Fe

SIFSIX-3-Fe is synthesized by solvothermal reaction of ferrous hexafluorosilicate, FeSiF_6_·6H_2_O, (1 mmol) with pyrazine, pyz, (2 mmol) in 20 ml methanol at 85 °C. A dark yellow powder was obtained after 3 days, collected from the Teflon bomb, and then washed with methanol. After the filtration of the yellow powder, the yellow filtrate was slowly evaporated to form yellow crystals of SIFSIX-3-Fe after 1 day.

### Synthesis of [Ni(pyz)_2_(SiF_6_)], SIFSIX-3-Ni

SIFSIX-3-Ni was prepared using the previously reported procedure^33^ by dissolving 10 mmol of pyrazine (pyz) and 5 mmol of NiSiF_6_·6H_2_O in 30 ml of methanol and heating at 75 °C for 3 days .

## Results and discussion

**Fig. 2:**
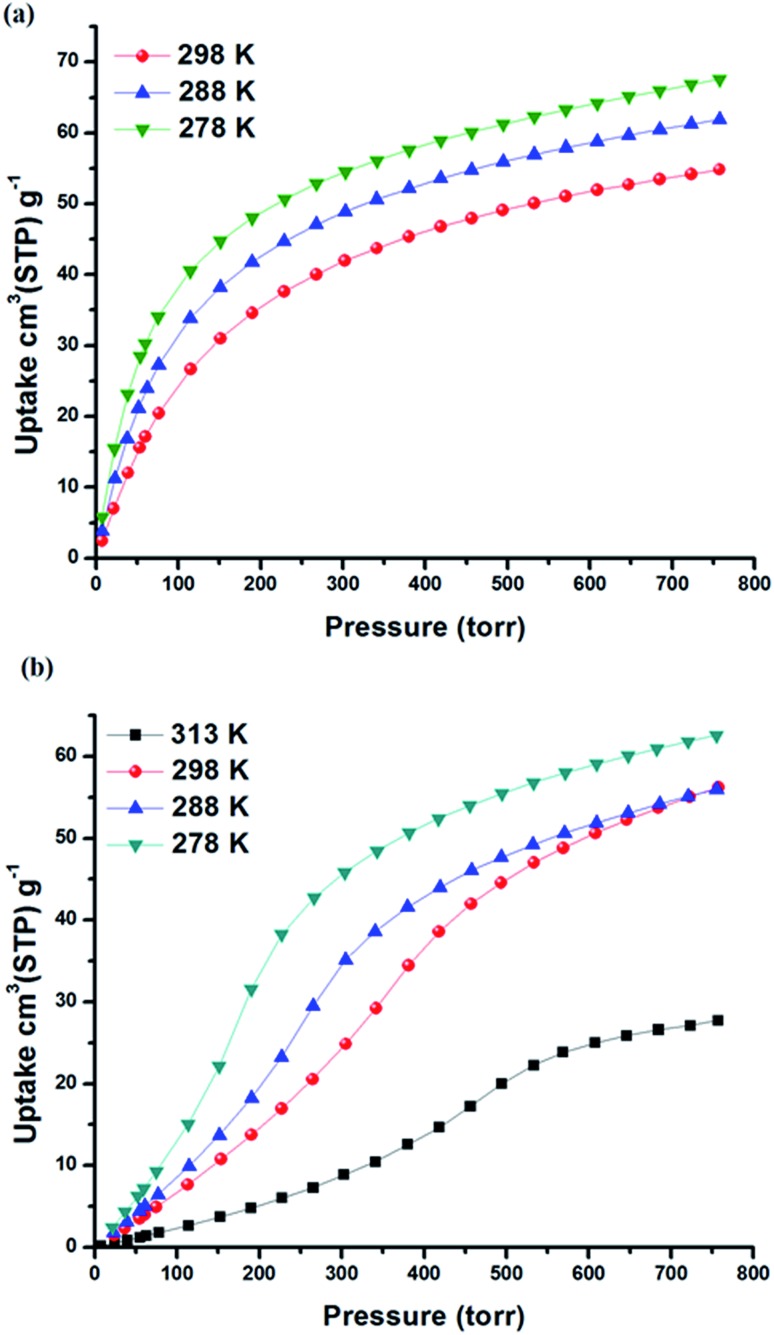
Xe adsorption isotherms collected at different temperatures for (a) SIFSIX-3-Fe and (b) SIFSIX-3-Ni.

The permanent porosity of SIFSIX-3-Fe was confirmed by N_2_ adsorption measurements at 77 K that revealed a Brunauer–Emmett–Teller (BET) surface area of 358 m^2^ g^–1^ (Fig. S2 in ESI†). Single component gas adsorption isotherms for Xe and Kr were collected at 298 K from 0–1 atm (Fig. 2 and S3 in ESI†). Xe uptake of SIFSIX-3-Fe at 1 atm and 298 K was found to be 54.9 cm^3^ STP g^–1^, whereas Kr uptake is 30.8 cm^3^ STP g^–1^. The sharp increase in Xe uptake in the low-pressure region reveals a high affinity of SIFSIX-3-Fe for Xe (30 cm^3^ STP g^–1^ at 0.1 bar) compared to other benchmark materials (see Fig. S4–S6 in ESI†).

**Fig. 3:**
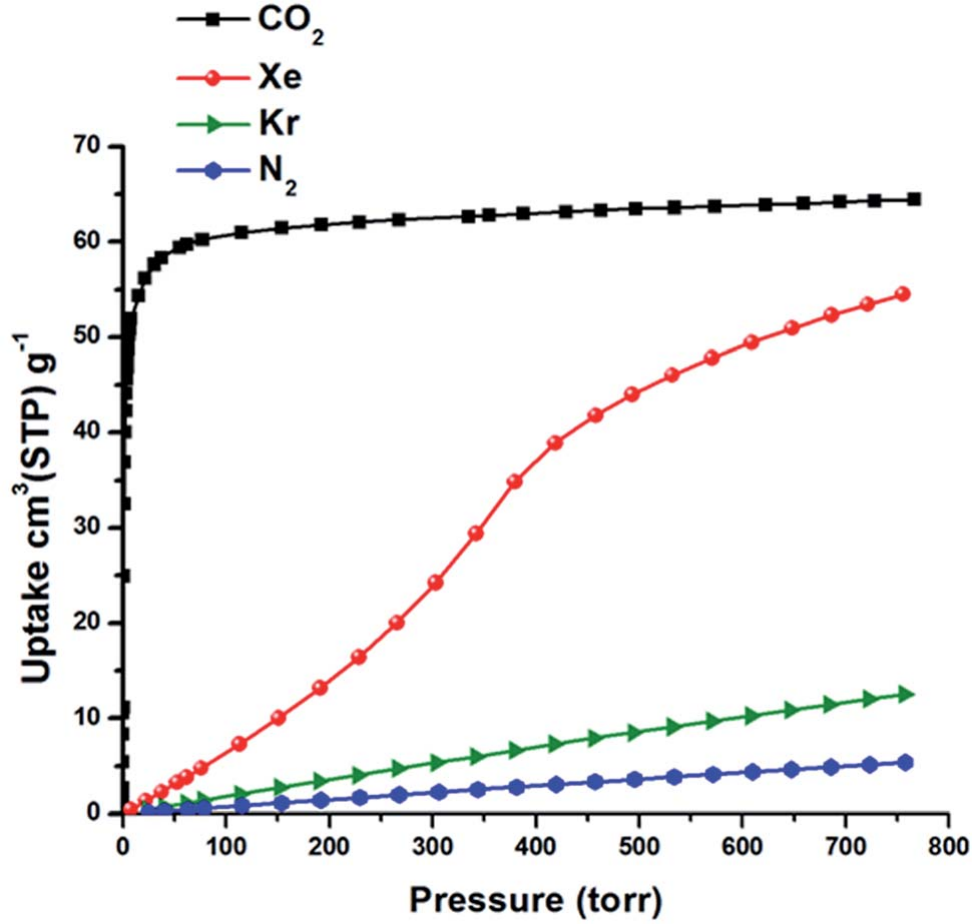
Single component gas adsorption isotherms in SIFSIX-3-Ni collected at 298 K.

The isosteric heat of adsorption (*Q*_st_) of Xe for SIFSIX-3-Fe was calculated (Viral equation) using adsorption data at 278, 288 and 298 K. The *Q*_st_ of Xe in SIFSIX-3-Fe was found to be 27.4 kJ mol^–1^ at infinite dilution (see Fig. S9–S12 in ESI†). To put this into perspective, the Xe uptake and *Q*_st_ at low pressure in SIFSIX-3-Fe is higher than NiDOBDC (22 kJ mol^–1^)^48^ and comparable to the porous organic cage CC3 (ref. 43) (31.3 kJ mol^–1^). However, *Q*_st_ of Xe is lower than in mmo topology nets (*Q*_st_ = –37.4 and –30.5 kJ mol^–1^ for CROFOUR-1-Ni and CROFOUR-2-Ni, respectively, at zero loading).^49^ SIFSIX-3-Ni exhibits a BET surface area (368 m^2^ g^–1^) and pore size (3.66 Å) similar to its Fe analogue. However, the single component adsorption isotherm of Xe in SIFSIX-3-Ni is qualitatively different from that of SIFSIX-3-Fe. At low pressures, the isotherm is convex and then transitions through an inflection point to concave, resembling a type V adsorption isotherm with no hysteresis (see Fig. 2b, 3 and S7–S8 in ESI†). Interestingly, the Kr adsorption isotherm shows no such inflection point, presumably because of its smaller kinetic diameter (see Fig. 3). Xe and Kr uptakes in SIFSIX-3-Ni of 56.2 and 12.6 cm^3^ STP g^–1^ were measured at 1 atm and 298 K, respectively. In addition, we studied the effect of temperature on the inflection point; as shown in Fig. 2b, the inflection point in the Xe isotherm in SIFSIX-3-Ni become more pronounced and shifts to lower pressures as temperature is decreased. The *Q*_st_ of Xe in SIFSIX-3-Ni at low coverage was found to be 18.9 kJ mol^–1^, lower than in its Fe analog (Fig. S9–S12 in ESI†). Nevertheless, the *Q*_st_ increases to 21 kJ mol^–1^ at moderate loadings, and SIFSIX-3-Ni exhibits nearly equivalent Xe uptake to that of SIFSIX-3-Fe at 1 atm (Fig. 2 and 3).

**Fig. 4:**
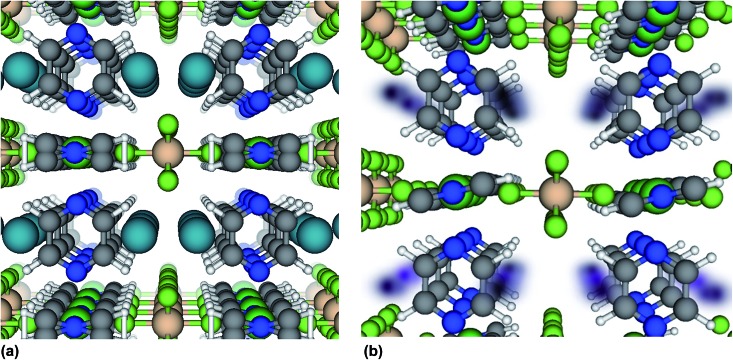
Location of adsorbed Xe atoms in SIFSIX-3-Ni from (a) powder X-ray diffraction studies (b) snapshots of Xe positions from molecular simulations of Xe adsorption at 0.2 bar. In (b), the darker color shows higher spatial probability density. Note that only one Xe will fit in each cage at any given time. The view is down the *c*-axis.

We now further address the question of why the Xe adsorption isotherm in SIFSIX-3-Ni exhibits an inflection point and why the isotherm in SIFSIX-3-Fe does not. Structural flexibility,^7,50^ adsorbate–adsorbate attractions,^51,52^ pore filling,^53^ capillary condensation,^54,55^ and commensurate-to-incommensurate adsorption transitions^56^ have been known to induce inflection points in adsorption isotherms. The location of the adsorbed Xe within SIFSIX-3-Ni was determined with *in situ* synchrotron-based PXRD (Fig. S23–S25†).^57^ Xe atoms reside in the center of the 1D channel along the *c* axis of the crystal lattice (see Fig. 4a). There is a slight expansion of both the *a*/*b* and *c*-axis upon Xe binding. According to the Xe–Xe Lennard-Jones potential^58^ and the van der Waals radius of Xe,^59^ prohibitively large repulsive forces would prevent two Xe atoms from occupying a single cage of SIFSIX-3-M at the positions observed from *in situ* XRD in Fig. 4a, precluding strong adsorbate–adsorbate attractions and imposing commensurate adsorption (one Xe atom per cage).

Inflection points were observed in the CO_2_ adsorption isotherms in [Co(HLdc)]·1.5MeOH·dioxane^30^ (hysteresis observed) and MIL-91(Al)^27^ (hysteresis not observed). In both of these studies, *in situ* XRD data indicated that the ligand in the CO_2_-loaded structure rotated from its orientation in the activated structure, and significant differences in the simulated CO_2_ adsorption isotherms in the two rigid hosts support that the inflection is a consequence of the twisting of the ligands. A similar ligand swing was observed in ZIF-8.^60^ To verify whether ligand rotation could also explain the inflection point observed here, we performed *in situ* PXRD measurement at beamline 17-BM-B at Advanced Photon Source (Argonne National Laboratory) on SIFSIX-3-Ni under three distinct environments: He-loaded, Xe-loaded, and under vacuum. The results showed that the pyz rings in all three cases had similar orientations in bulk, where each ring is rotated at around ±16 degrees about the respective crystal axis (Fig. S16, S23–S25 and Table S1†). This tilt of the rings about the crystallographic planes observed by our XRD study is consistent with our DFT energy minimized SIFSIX-3-Ni structure (see ESI†) and with theoretical and experimental studies of a cousin, SIFSIX-3-Zn.^61^ Note that *in situ* XRD does not provide conclusive evidence of ring rotation, however.

Here, we propose and provide computational evidence that the inflection point in the Xe adsorption isotherm in SIFSIX-3-Ni is due to a very different structural phenomenon: a subtle transition in the rotational orientations of the pyrazine rings. In this transition, the pyrazine rings gradually organize their rotational configurations to better accommodate Xe as guests as Xe loading increases. Note this hypothesis does not conflict with the synchrotron based *in situ* powder XRD observations, which only indicate the ring orientations in bulk: individual rings can still flip between +16 or –16 configurations without altering the bulk structure. This ±16 degree rotation enables CH···F interactions of *ca.* 3.2–3.3 Å in this and related structures but is different to that observed in CO_2_ loaded SIFSIX-3-M, where the pyz rings are parallel to the *c*-axis.^33,37^ With respect to a particular cage of interest, we will refer to the rotation in which the plane of the pyrazine ring faces into the cage as the “IN” configuration, and the rotation in which the plane of the pyrazine ring faces out of the cage as the “OUT” configuration.

**Fig. 5:**
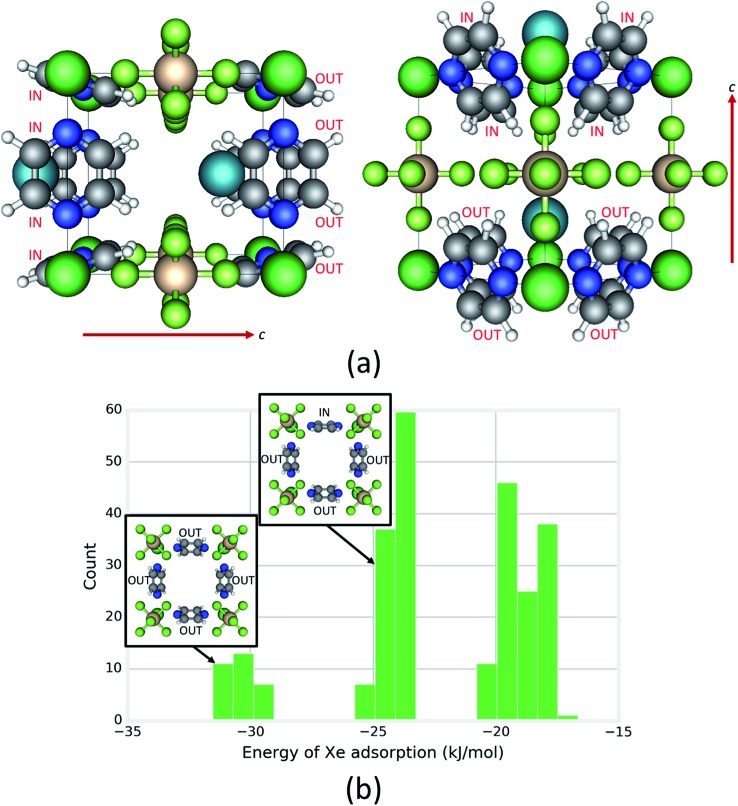
Investigating the influence of the rotational configurations of pyrazine rings on xenon adsorption. (a) Two different views (*c*-axis marked) of the one of the 2^8^ possible rotational configurations of rings in a cage that yields the most favorable Xe energy of adsorption. Configurations of pyz rings are labeled as IN or OUT. The cage is defined by the box formed by the black lines. (b) The distribution of Xe adsorption energies among the 2^8^ possible rotational configurations of rings in a cage. Note the three distinct clusters. The two insets show the characteristic 4-ring configuration contained in the respective cluster, with a view down the *c*-axis.

Under the constraint that each pyrazine ring can adopt either an IN or OUT configuration with respect to a cage, each cage can adopt one of 2^8^ = 256 possible states (see Fig. 5a; black box defines a cage). We constructed rigid cages of each of these states *in silico* using unit cell parameters taken from DFT-optimized structures and calculated the ensemble average energy of Xe adsorption in each cage using a classical molecular model (see ESI for details Fig. S13–S17†). The distribution of Xe adsorption energies in these 256 cage states of SIFSIX-3-Ni in Fig. 5b shows that the orientation of the pyrazine rings has a significant influence on the Xe adsorption energy. The cage with the most favorable Xe energy of adsorption (Fig. 5a) exhibits a set of four OUT rings on one half of the cage and four IN rings on the other half; a Xe at its minimum energy position in this type of cage is “hugged” by the four OUT rings with their planes oriented more tangential to the Xe atom. Note the three distinct clusters in the distribution in Fig. 5b. All configurations in the cluster with the most favorable (lowest) Xe energy of adsorption exhibit a set of 4 OUT rings as in the minimum energy configuration (see inset of Fig. 5b); this 4-ring configuration is not seen in the two other clusters with higher energies. All configurations in the intermediate cluster contain a 4-ring configuration with three OUT rings (see inset of Fig. 5b). In contrast, the distribution of Xe adsorption energies in the Fe analogue spans a smaller range of energies and displays only two, less distinct clusters (see Fig. S18†), showing that the rotational configurations of the rings have a lesser influence on the host–guest interaction in the Fe analogue because of its larger cage size. The rotational configurations of the rings that yield the most favorable Xe adsorption energy for the Fe analogue are analogous to Fig. 5a.

**Fig. 6:**
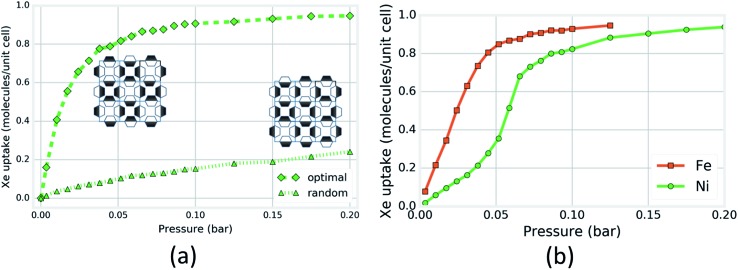
Computational support for the induction of an inflection point in the Xe isotherm of SIFSIX-3-Ni by organization of pyz ring rotational configurations. (a) Simulated Xe adsorption isotherms in two rigid SIFSIX-3-Ni structures, one with each cage exhibiting the configuration shown in Fig. 5a and the other with each ring configuration chosen at random. Insets are caricatures of organized (left) and random (right) ring configurations looking down the *c*-axis; a box represents a channel and shading denotes rotational conformation. (b) Simulated Xe adsorption isotherms in SIFSIX-3-Ni and -Fe when each pyz ring is allowed to freely flip between +16 and –16 degree tilts about their respective crystallographic planes.

Next, we investigated the magnitude of the effect that the rotational configurations of the rings can have on the simulated Xe adsorption isotherms. We constructed two rigid-host structures with 16 cages: the rotational configuration of each ring in the first structure is chosen at random; in the second structure, the rotational configurations are chosen so that each cage looks as in Fig. 5a, the cage that achieves the optimal energy of Xe adsorption. Each cage in the structure can achieve this configuration by aligning its pyrazine rings down the *c*-axis and forming a chessboard pattern from the view normal to the *c*-axis (see Fig. S19†). The four OUT rings on one side of this optimal cage construct an optimal binding site for Xe while the four IN rings on the other side provide the neighboring cage with four OUT rings for an optimal Xe binding site. Fig. 6a shows that the Xe adsorption isotherm in the structure with organized ring configurations saturates at a lower pressure as a consequence of its more favorable guest–host interaction. For SIFSIX-3-Fe, however, the difference between the two rigid host isotherms is less drastic than for the Ni analogue (see Fig. S18†) (Fig. 6a).

The energies of the SIFSIX-3-Fe and SIFSIX-3-Ni crystal structures as calculated using VASP^62,63^ indicate that the minimum energy configuration for a vacant corridor corresponds to the IN–OUT–IN–OUT configuration, shown to be suboptimal for Xe adsorption in the simulations described above. Introduction of one Xe atom per cage into the SIFSIX-3-Fe and SIFSIX-3-Ni systems, however, introduces new energetic effects, which cause the minimum energy configuration to shift to the more favorable OUT–OUT–OUT–OUT conformation next to each adsorbed Xe atom. This is consistent with the addition of Xe gradually reorganizing the rotational configurations of the pyz rings, causing the structure to eventually become a more receptive adsorbent.

Our hypothesis is that, under vacuum, the rings are exploring their microstates by flipping between ±16 degrees, adopting approximately random/uncorrelated rotations with respect to one another down the *c*-axis, while primarily residing in IN–OUT–IN–OUT configurations in the *a*/*b* plane. As Xe atoms are introduced into the structure, the rotational configurations of the rings gradually rearrange to the OUT–OUT–OUT–OUT configuration with organization along the *c*-axis to achieve more favorable guest–host interactions as in Fig. 5a. One can envision that, in the process of the gradual structural transition, the Xe adsorption isotherm transitions from the bottom curve in Fig. 6a to the top curve through an inflection point.

To test if freely flipping pyrazine rings can induce an inflection point in the Xe adsorption isotherm of SIFSIX-3-Ni, we simulated Xe adsorption in the grand canonical (μVT) ensemble while allowing each pyrazine ring to flip between the two rotational configurations with no intra-host energetic penalty (see ESI† for details). The simulated Xe adsorption isotherm in SIFSIX-3-Ni with freely flipping pyrazine rings displays a pronounced inflection point (Fig. 6b) as the rings organize (Fig. S16†) to achieve a more favorable guest–host interaction (Fig. S20†). The simulated Xe adsorption isotherm in SIFSIX-3-Fe with ring flipping does not exhibit an inflection point as a consequence of the smaller effect that the rotational configurations of the rings have on the Xe adsorption energy. In concordance with the experimental results in Fig. 3, the simulated adsorption isotherms for Kr and CH_4_ in SIFSIX-3-Ni with flipping rings do not exhibit an inflection point (Fig. S21–S22†), indicating that the presence of an inflection point due to flipping rings is sensitive to the match between the adsorbate and cage size.

Notably, a more gradual ring ordering effect similar to the one postulated here is reported for CO_2_ adsorption in SIFSIX-1-Cu, [Cu(bpy)_2_SiF_6_] (bpy = 4,4′-bipyridine).^64^ It was observed that simulations of CO_2_ adsorption in a rigid structure corresponding to the lowest energy ring rotational conformation drastically underestimated the experimental adsorption due to orientational constraints on the adsorbed CO_2_ molecules, while the simulated uptakes in various structures with higher energy ring orientations produced results that were in good agreement with experiment. We speculate that, relatedly, pyrazine ring rotation may be involved in a suspected phase change in SIFSIX-3-Zn^32,65^ and gate opening in Fe(py)_2_[Pt(CN)_4_].^24^

According to our simulations, the inflection point in the Xe isotherm in SIFSIX-3-Ni broadens and shifts to higher pressures with increasing temperature (Fig. S21†). The broadening is consistent with the notion that the rings have an entropic incentive to dynamically flip to explore their microstates; as Xe adsorbs, the rings organize to achieve a greater host–guest interaction at the expense of entropy. At higher temperatures, entropy begins to dominate the free energy, and the inflection broadens. The experimentally measured isotherms at 313 K, 298 K, 288 K, 275 K and 195 K affirm the inflection point shifting toward higher pressures and broadening as the temperature increases (see Fig. 2b and S8b†). Remarkably, the inflection point occurs at ∼0.5 Xe adsorbates per cage. We postulate that this is not a coincidence; when 1/2 of the cages are occupied by Xe, the rings cannot adopt orientations independent of one another without interfering with a host–Xe interaction, requiring a long-range organization. This also explains why the inflection point shifts to higher pressures as the temperature increases.

Interestingly, Kanoo *et al*.^61^ found that SIFSIX-3-Zn adsorbs more carbon dioxide at 298 K than at 195 K. Consistent with our XRD studies and DFT calculations, they found the pyz rings in SIFSIX-3-Zn adopt 17° tilts about their respective crystallographic planes. Furthermore, their Raman spectroscopy studies implied that the structure becomes more symmetric upon the adsorption of CO_2_ , likely due to changes in the alignment of the pyz rings. Their spectroscopic data in the SIFSIX-3-Zn analogue is consistent with our hypothesis of a disordered to ordered transition of the pyz rings in SIFSIX-3-Ni as xenon adsorbs.

## Conclusions

In summary, we report a new isostructural porous pillared square grid net, SIFSIX-3-Fe, that exhibits high isosteric heat of adsorption of Xe and preferential adsorption of Xe over Kr. We attribute this behavior to the optimally tuned pore size that is commensurate with the size of Xe atom. An inflection in the Xe adsorption isotherm in SIFSIX-3-Ni arises, and we attribute this behavior to a disordered to ordered transition of the rotational configurations of the pyrazine rings as opposed to other phenomena such as guest–guest interactions or breathing. In this transition, the rings organize their rotational configurations to achieve a greater guest–host interaction. To our knowledge, such dynamic behavior has not been suggested previously as the origin of an inflection in gas adsorption. Our understanding is a step towards the lofty goal of engineering MOFs with moving parts to harness these dynamics for applications in gas sensing and separations, drug delivery, and catalysis.
