# Supplementary material for: Effect of ring rotation upon gas adsorption in SIFSIX-3-M (M = Fe, Ni) pillared square grid networks
Source: Chem Sci. 2016 Dec 19;8(3):2373–80. doi: 10.1039/c6sc05012c (PMC5364996; doi:10.1039/c6sc05012c)
Supplement: Supplementary file 1 [file SC-008-C6SC05012C-s001.pdf]

Electronic Supplementary Material (ESI) for Chemical Science.
This journal is © The Royal Society of Chemistry 2016

Supporting Information

Effect of Ring Rotation upon Gas Adsorption in SIFSIX-3-M (M = Fe, Ni) Pillared Square Grid Networks

Sameh K. Elsaidi, Mona H. Mohamed, Cory M. Simon, Efrem Braun, Tony Pham, Katherine A. Forrest, Wenqian Xu, Debasis Banerjee, Brian Space, Michael J. Zaworotko and Praveen K. Thallapally

Methods:

Synthesis of [Fe(pz)$_2$(SiF$_6$)$_2$], SIFSIX-3-Fe:

SIFSIX-3-Fe is synthesized by solvothermal reaction of ferrous hexafluorosilicate, $FeSiF_6.6H_2O$, (1 mmol, 0.31 g) with pyrazine, pz, (2 mmol, 0.16 g) in 20 ml methanol at 85 °C. Dark yellow powder was thereby afforded after 3 days then collected from the Teflon bomb and washed by methanol. After the filtration of the yellow powder, the yellow filtrate was slowly evaporated to form yellow crystals of SIFSIX-3-Fe after 1 day. Single-crystal X-ray crystallography revealed that [Fe(pz)$_2$(SiF$_6$)$_2$] crystallizes in the tetragonal space group P 4/m m m (a=b=7.1831(11) Å, c=7.5839(12) Å).

SIFSIX-3-Fe sample activation:

The as-synthesized crystals of SIFSIX-3-Fe were exchanged with methanol for 3 days (2 times/day) prior the activation. The resulting solid was filtered and evacuated at 75 ^{o}C for 15 h under dynamic pressure (<5µm Hg).

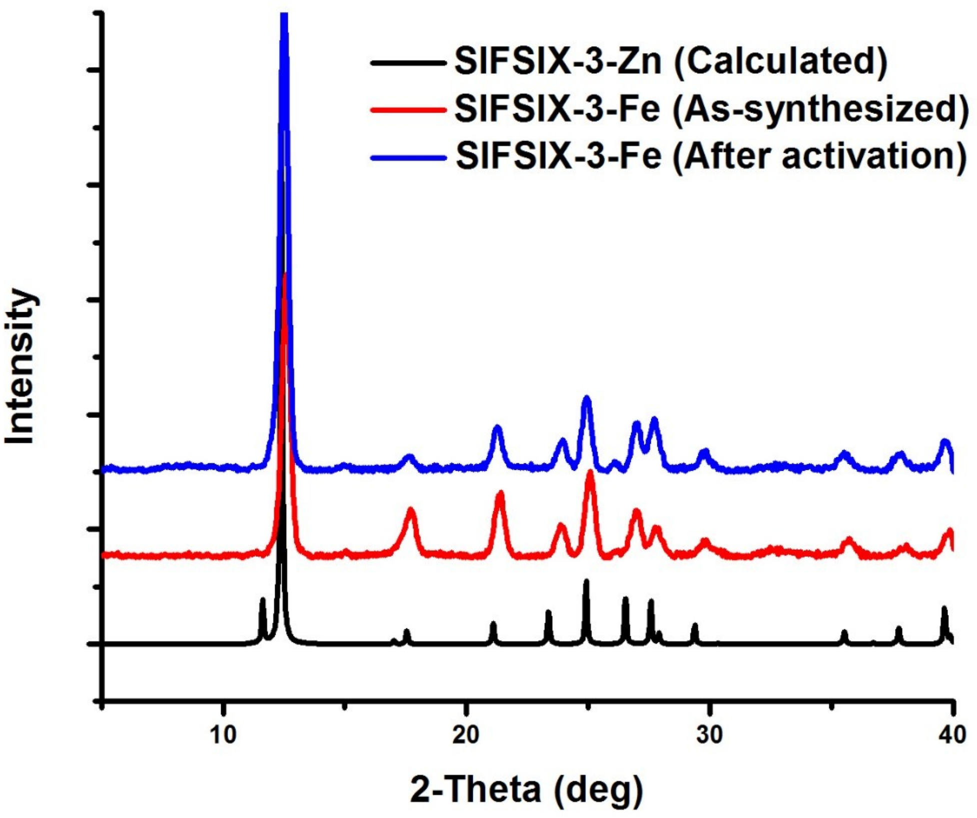

Figure S1. Powder X-ray diffraction patterns (PXRD) of SIFSIX-3-Fe.

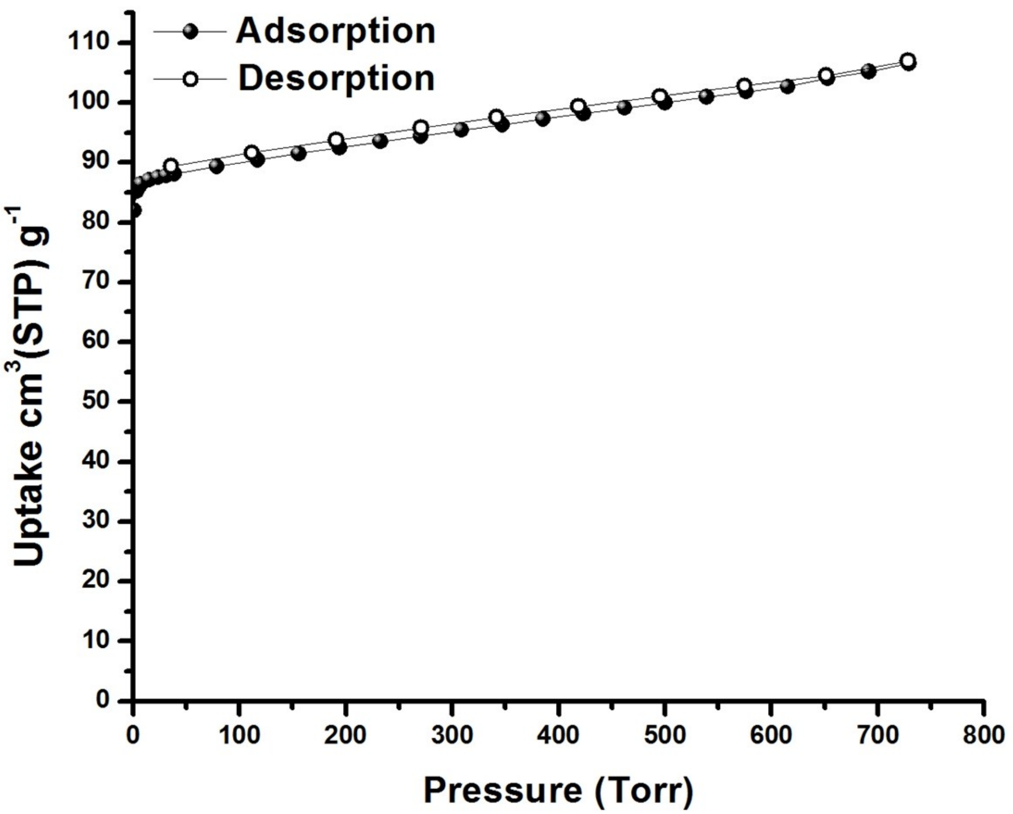

Figure S2. Single component N_2 isotherm collected at 77 K for SIFSIX-3-Fe.

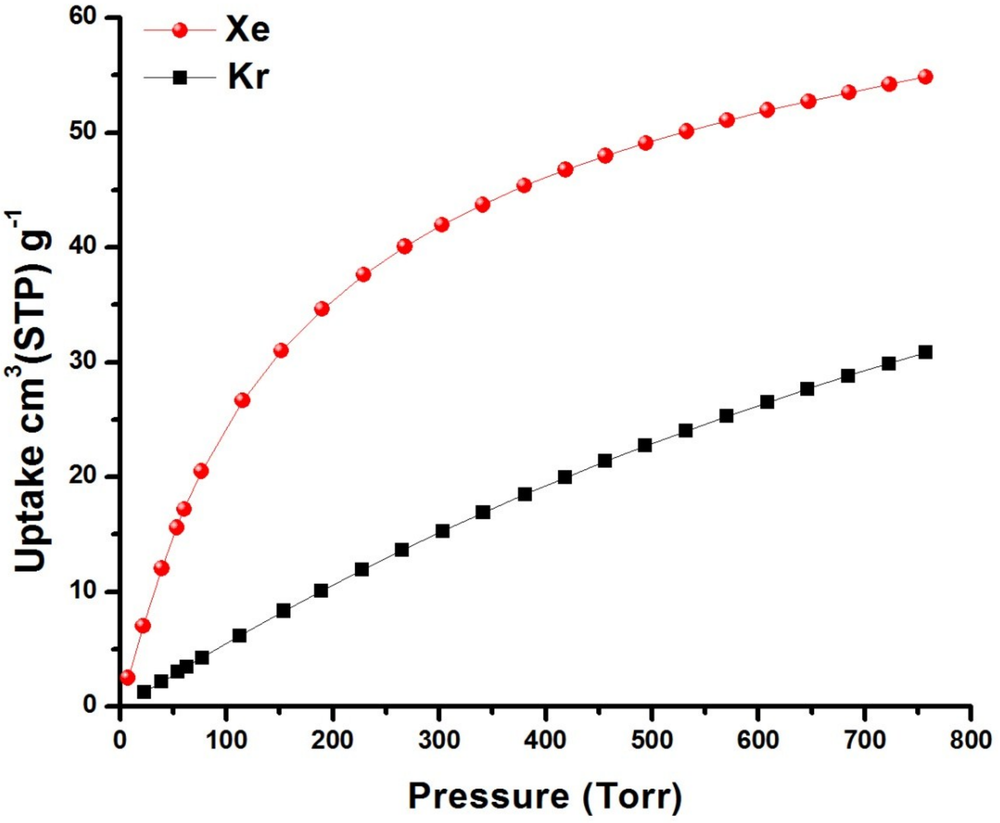

Figure S3. Single component Xe and Kr sorption isotherms for SIFSIX-3-Fe measured at 298 K.

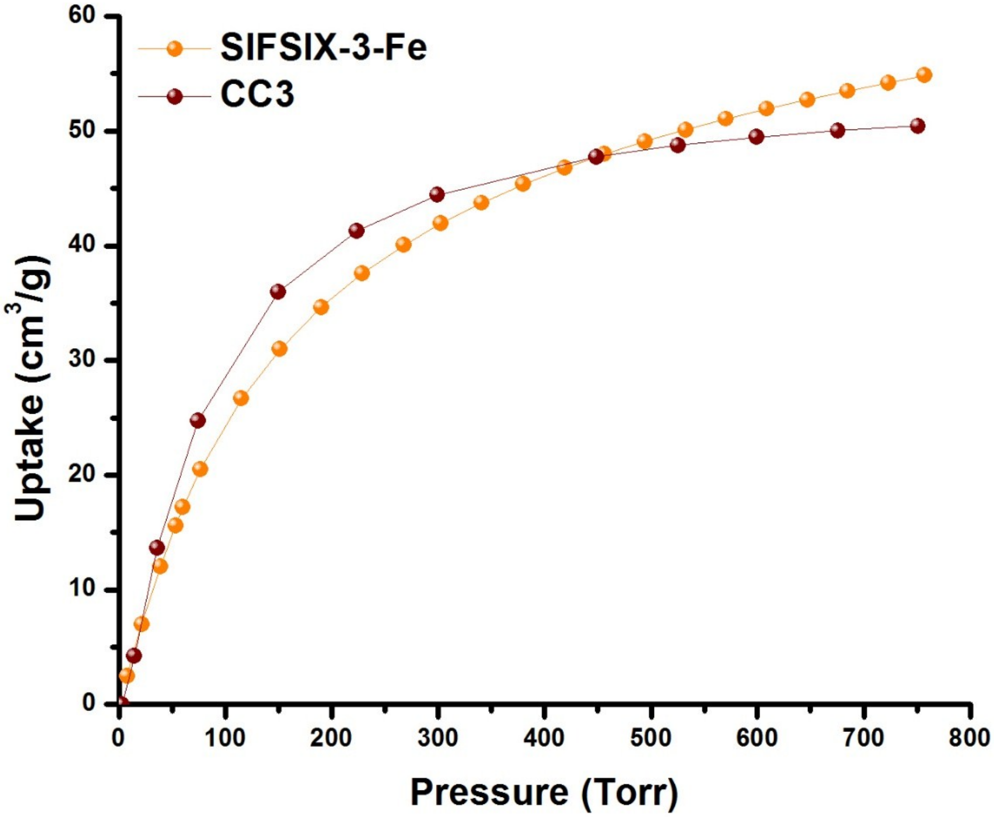

Figure S4. Single component Xe sorption isotherms for SIFSIX-3-Fe and CC3 measured at 298 and (0-760) torr.

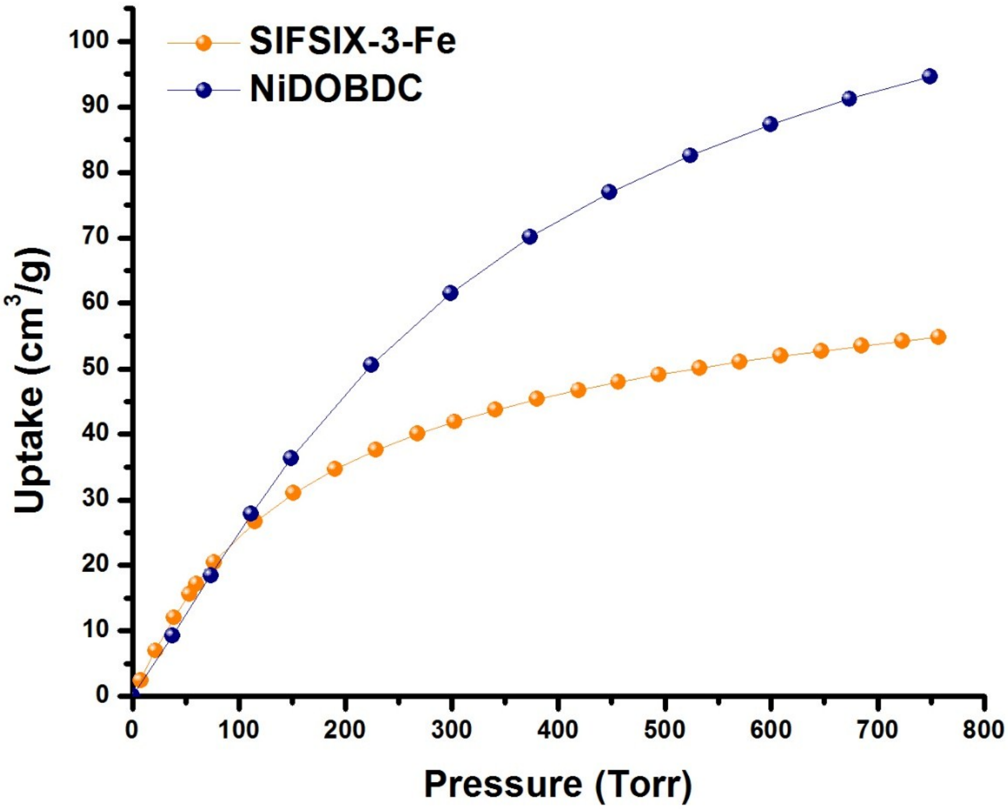

Figure S5. Single component Xe sorption isotherms for SIFSIX-3-Fe and NiDOBDC measured at 298 and (0-760) torr.

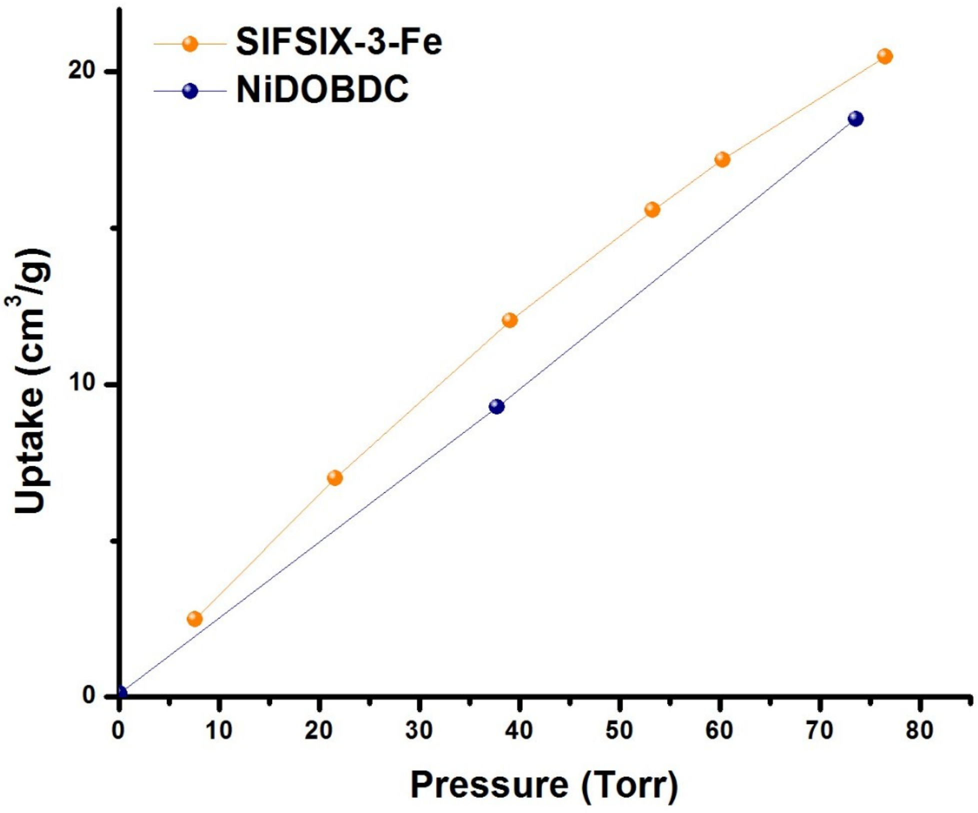

Figure S6. Single component Xe sorption isotherms for SIFSIX-3-Fe and NiDOBDC measured at 298 and (0-80) torr.

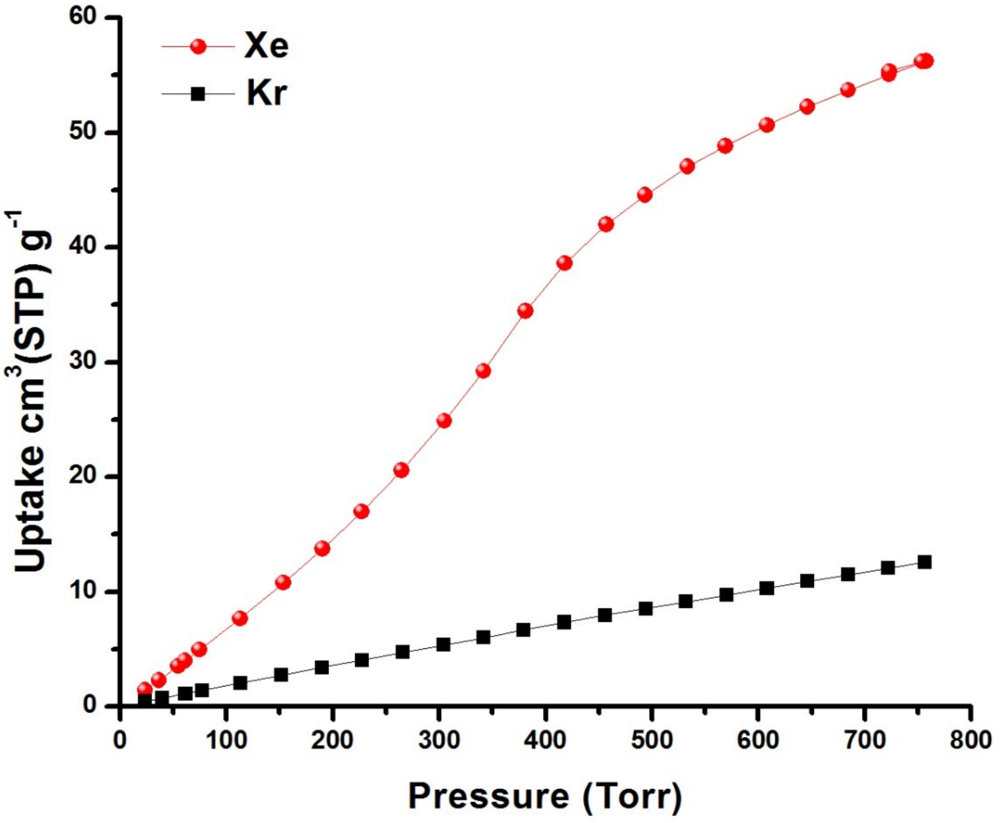

Figure S7. Single component Xe and Kr sorption isotherms for SIFSIX-3-Ni measured at 298 K.

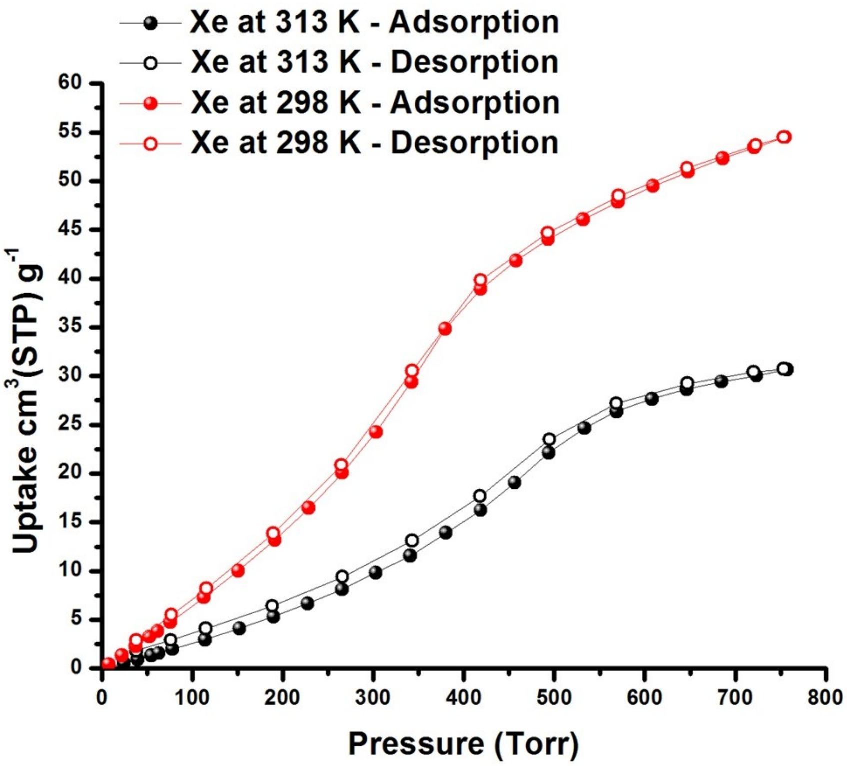

(a)

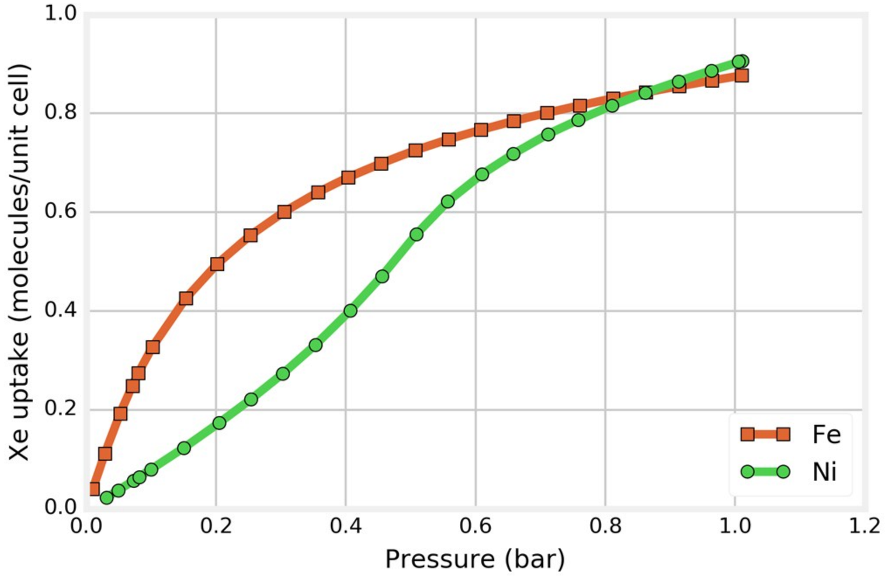

(b)

Figure S8. (a) Reversible Xe sorption isotherms for SIFSIX-3-Ni measured at 298 K and 313 K and (b) Xe adsorption isotherms for SIFSIX-3-Ni and SIFSIX-3-Fe measured at 298 K.

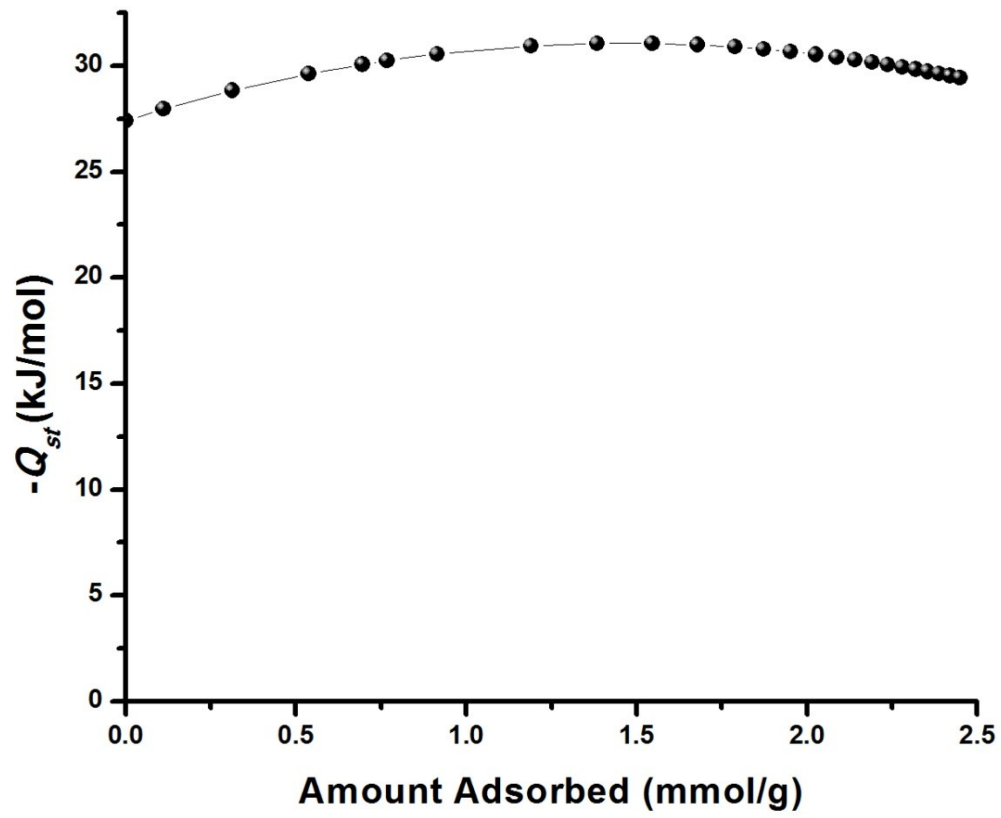

Figure S9. Xe isosteric heats of adsorption (Q_{st}) for SIFSIX-3-Fe.

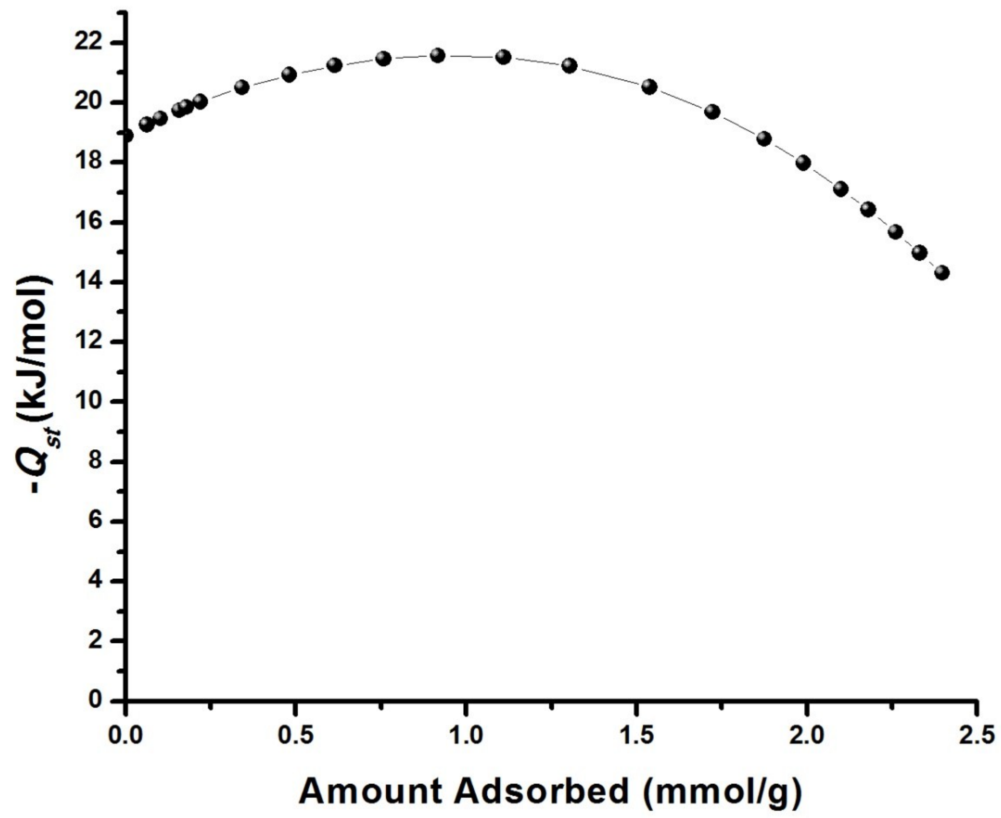

Figure S10. Xe isosteric heats of adsorption (Q_{st}) for SIFSIX-3-Ni.

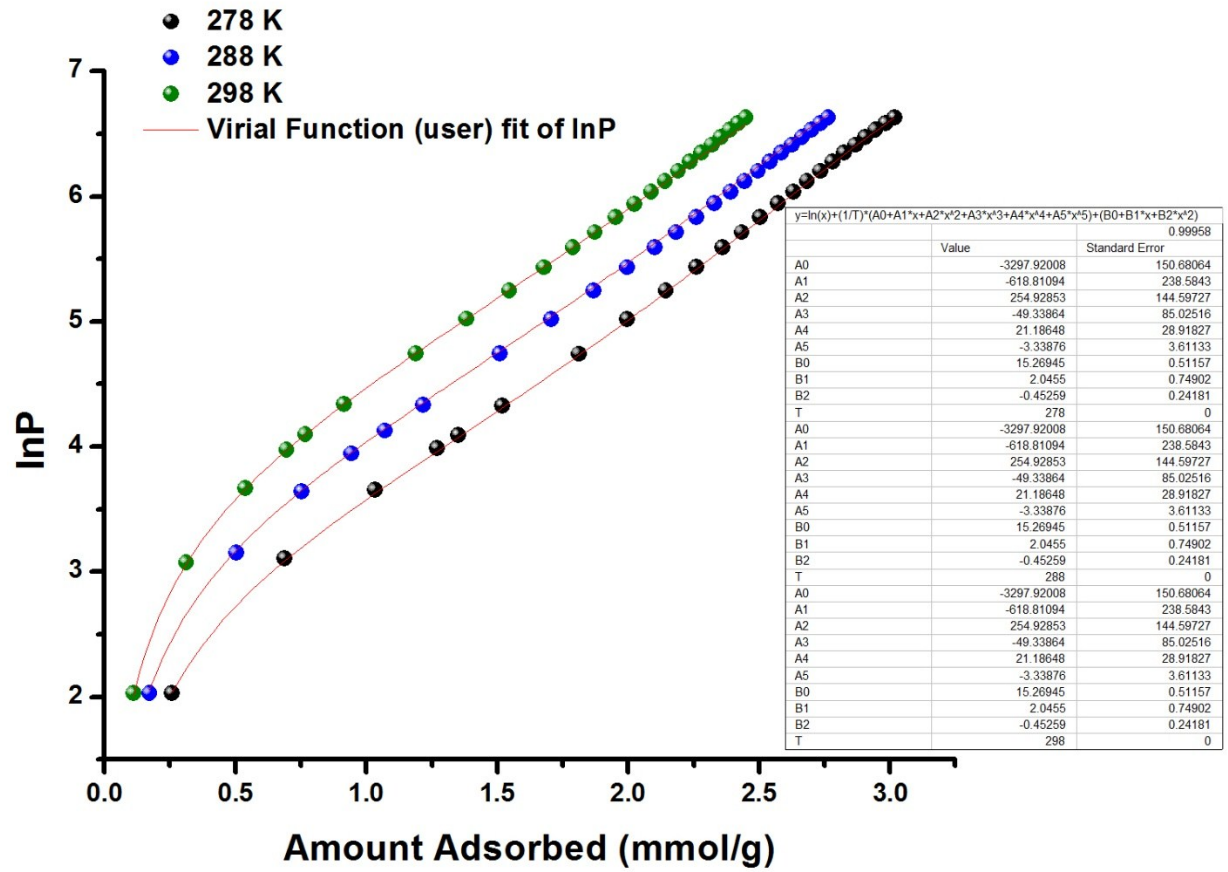

y=ln(x)+(1/T)*(A0+A1*x+A2*x^2+A3*x^3+A4*x^4+A5*x^5)+(B0+B1*x+B2*x^2)		
		0.99958
	Value	Standard Error
A0	-3297.92008	150.68064
A1	-618.81094	238.5843
A2	254.92853	144.59727
A3	-49.33864	85.02516
A4	21.18648	28.91827
A5	-3.33876	3.61133
B0	15.26945	0.51157
B1	2.0455	0.74902
B2	-0.45259	0.24181
T	278	0
A0	-3297.92008	150.68064
A1	-618.81094	238.5843
A2	254.92853	144.59727
A3	-49.33864	85.02516
A4	21.18648	28.91827
A5	-3.33876	3.61133
B0	15.26945	0.51157
B1	2.0455	0.74902
B2	-0.45259	0.24181
T	288	0
A0	-3297.92008	150.68064
A1	-618.81094	238.5843
A2	254.92853	144.59727
A3	-49.33864	85.02516
A4	21.18648	28.91827
A5	-3.33876	3.61133
B0	15.26945	0.51157
B1	2.0455	0.74902
B2	-0.45259	0.24181
T	298	0

Figure S11. Xe adsorption isotherms of SIFSIX-3-Fe at 278 K, 288 K and 298 K fitted using Virial equation.

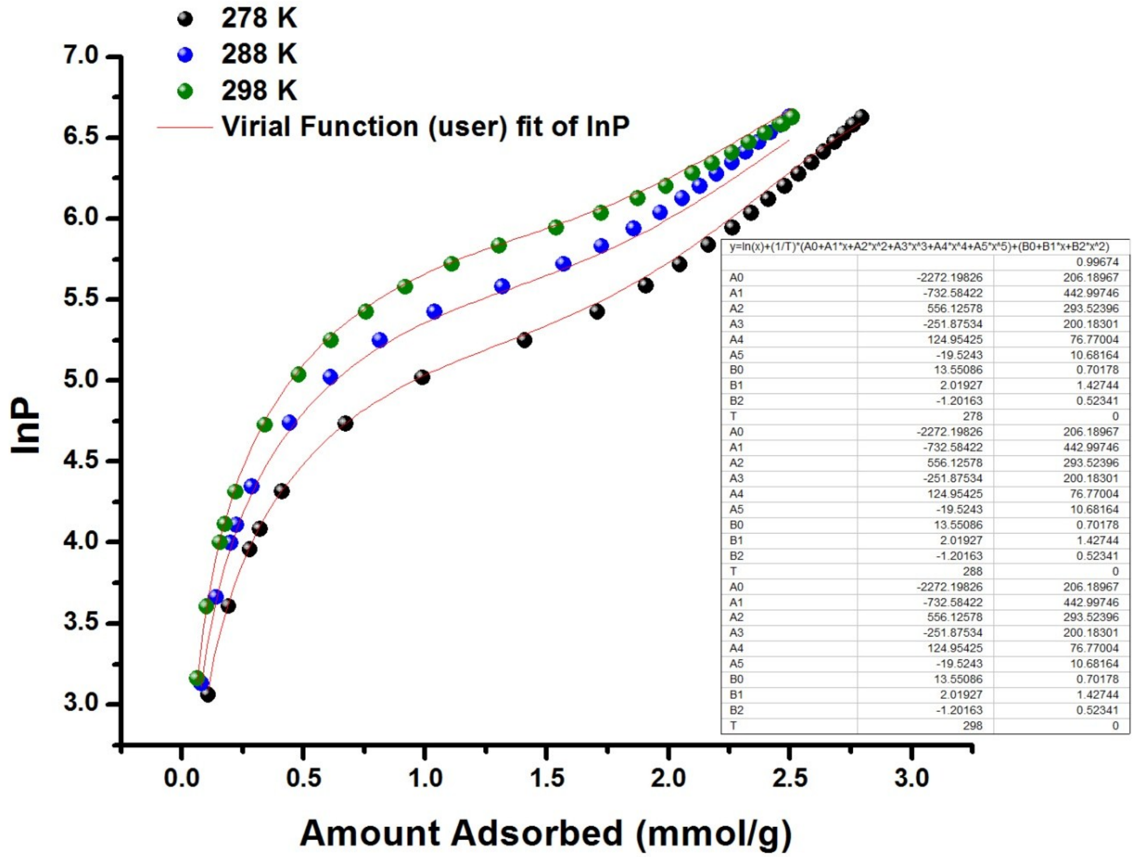

Figure S12. Xe adsorption isotherms of SIFSIX-3-Ni at 278 K, 288 K and 298 K fitted using Virial equation.

Molecular simulations

We model the energetics of the interactions between Xe and the atoms of the MOF (and other Xe atoms) with Lennard-Jones potentials. Parameters are taken from Ref. [1] for Xe, the TraPPE force field[2] for pyrazine (parameters fit to pyrazine critical data), the Dreiding force field[3] for SiF_6 and Fe, and the Universal Force Field[4] for Ni, which was not available in Dreiding. Parameters for cross-interactions are then obtained from Lorentz-Berthelot mixing rules. We use simple truncation of the potentials beyond a cutoff of 12.5 Å.

During our simulations, we apply periodic boundary conditions to mimic a crystal of infinite extent. We simulate adsorption in a supercell consisting of four cages in each of the a, b, and c directions in order to apply the nearest image convention for Xe-Xe interactions and capture a long enough length scale to observe long-range order in the rotational configurations of the pyrazine rings. The MOF structure is modeled as rigid, except for our allowing of the pyrazine rings to adopt either a +/- 16 degree rotation about their respective crystal axis as observed both in the DFT and XRD structures.

We consider an adsorbed phase of Xe in a 4×4×4 super-cell MOF in equilibrium with a bulk gas phase of Xe. We then perform Monte Carlo simulations of the grand-canonical ensemble,[5] where chemical potential of the bulk Xe gas, volume of MOF, and temperature are fixed. We relate the chemical potential of Xe to pressure with the ideal gas law.

To model the rotational degrees of freedom of the pyrazine rings, we allow each pyrazine ring in the MOF to exist in one of two states: rotated at + 16 or -16 degrees about the respective crystal axis. All microstates of the MOF are shown at once in Fig S13, resembling the disordered structure seen from the XRD data. During our Monte Carlo simulations, to model the rotational degrees of freedom of the pyrazine rings, we introduce an additional Markov chain move: to choose a ring at random and propose to flip it. The creation of this move was inspired by Coudert *et al*,[6] with flipping rings in SIFSIX-3-Ni falling under the category of intra-framework dynamics. We then calculate the energy of that pyrazine ring with the guest Xe adsorbate atoms in the system when it is in a +16 and -16 degree rotation. As an approximation, we take the intra-host energy differences between different pyrazine rotational configurations to be zero. We

accept the proposal to flip this ring with probability $min(1, e^{-\beta\Delta E})$, where ΔE is the difference in pyrazine-Xe energy between the final and initial state.

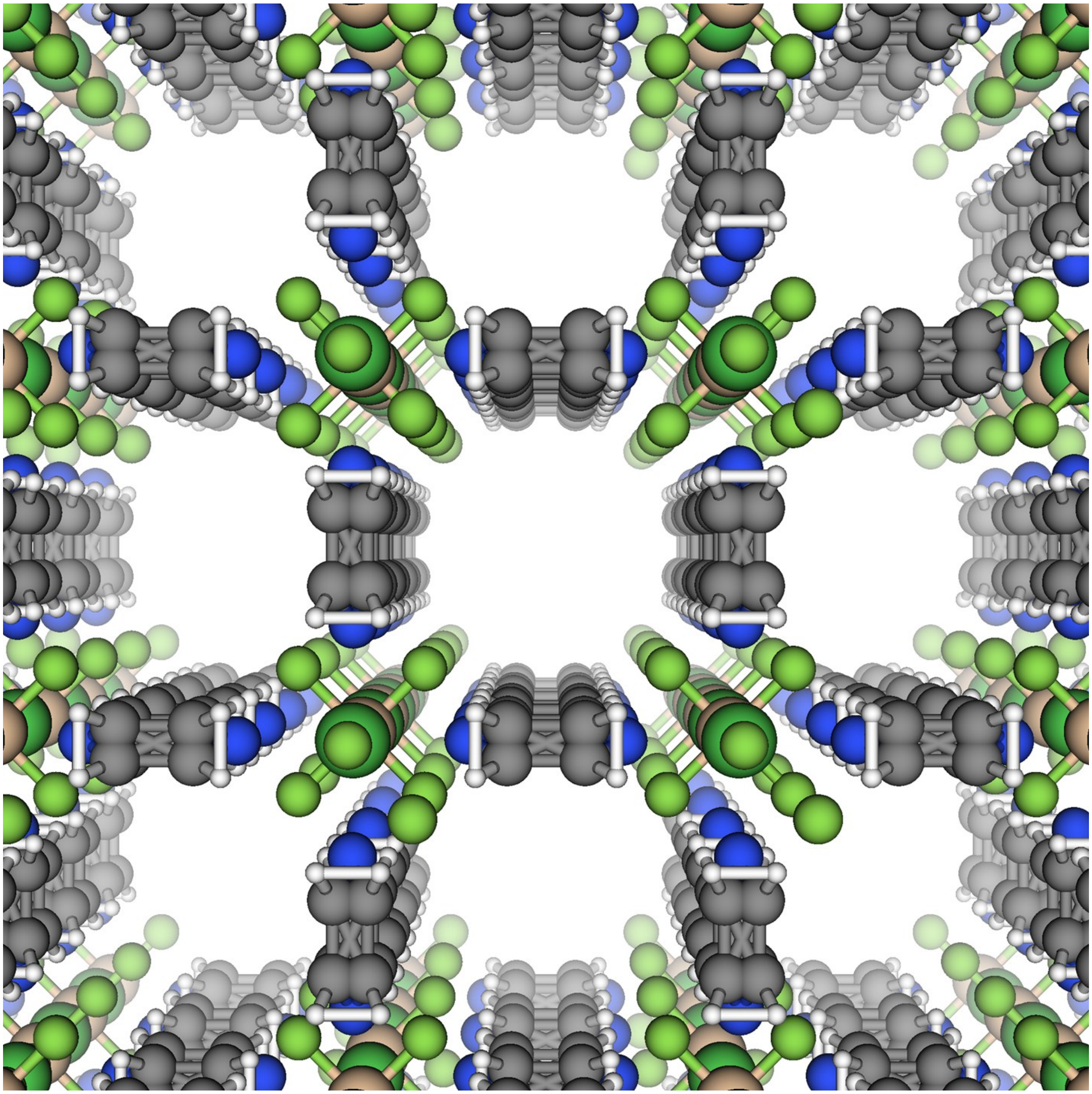

Figure S13. All microstates of SIFSIX-3-Ni MOF shown at once; each ring can be rotated either +16 or -16 degrees about its respective crystal axis.

We utilize 175,000 cycles for equilibration and 175,000 cycles for sampling statistics, where a cycle is defined as the minimum of {20, n}, where n is the number of Xe adsorbates in the system. We choose a Markov chain transition as a particle translation, particle exchange (insertion or deletion with equal probability), or ring flip with probability 0.2, 0.5, and 0.3, respectively.

Order parameters to characterize ring organization

By visual inspection of snapshots of the pyrazine (pyz) ring configurations in SIFSIX-3-Ni during the grand-canonical Monte Carlo simulations both before and after the inflection point, the rings appear to be randomly oriented at low pressures and organized to resemble the structure shown in Fig S19 after the inflection. Thus, this is a disorder to order transition of the pyz rings in SIFSIX-3-Ni. At low chemical potential, the rings entropically prefer to explore their microstates; as the chemical potential is increased, the rings organize (at an entropic cost) to afford the Xe a greater energetic interaction with the host. We now quantify the organization of the pyz rings during our grand canonical Monte Carlo simulations of Xe adsorption in SIFSIX-3-Ni with flipping pyz rings as the chemical potential increases. In the structure where each cage has the optimal Xe energy of adsorption (Fig S19), the rings are aligned (the same rotation) along the *c*-axis and in a chessboard-like pattern along the *ab*-plane. Thus, we must define two order parameters: one that quantifies order along the *c*-axis, and the other than quantifies order in the *ab*-plane.

Alignment of rings along the *c*-axis

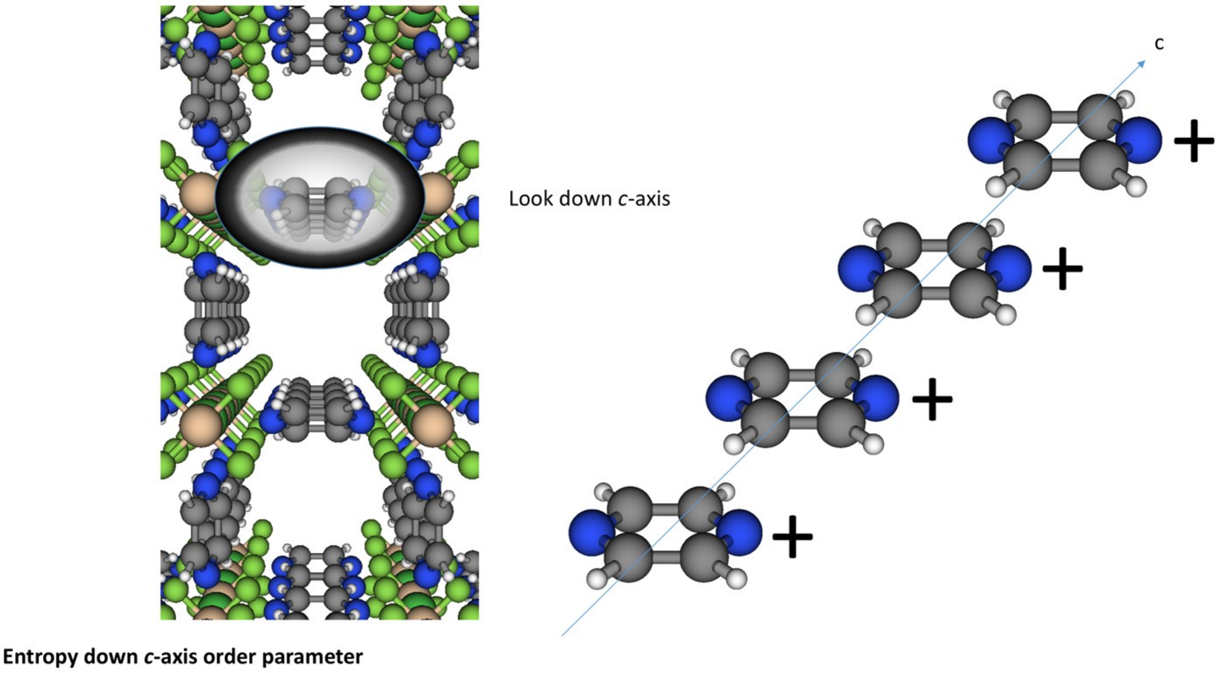

Figure S14. Cartoon depicting rings of concern when computing the entropic order parameter of rings down a line parallel to the *c*-axis.

Let p_+ and p_- be the proportion of rings rotated in the + and - direction, respectively, down a set of rings along a line parallel to the *c*-axis. Here, our order parameter is defined as the average *entropy* as one looks at each row of 4 rings down the channel (*c*-axis): $\langle -p_+ log(p_+) - p_- log(p_-) \rangle$

The entropy order parameter is at most ~0.69 when the rings are randomly oriented, $p_+ = p_- = \frac{1}{2}$, and, when the rings are all in the same rotation, the entropy order parameter is zero.

Formation of a chessboard pattern in the rings intersecting *ab*-planes

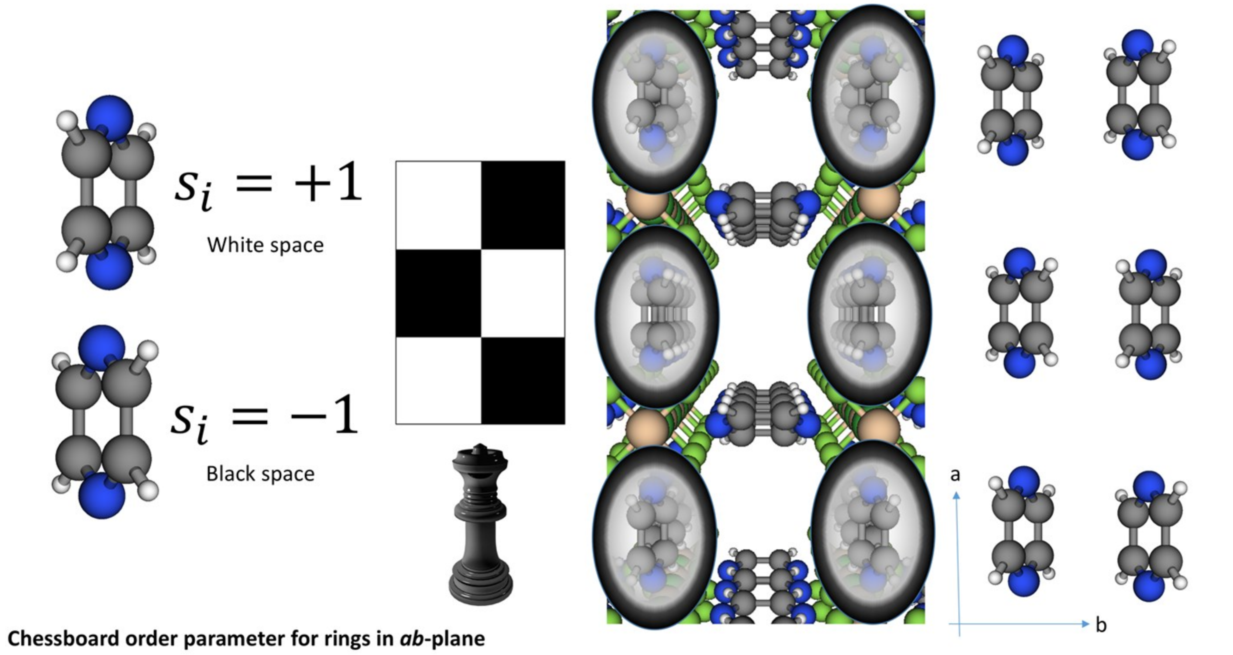

Figure S15. Cartoon depicting rings of concern when computing the energetic chessboard order parameter of rings in the *ab*-plane (the analogy is that black is a +16 degree rotation while white is a -16 degree rotation). Highlighted here is one of the two groups of rings in the *ab*-plane for which this order parameter is calculated; the computation for the other group of rings is analogous. Each group is defined by the plane to which the pyrazine faces are more tangential: the *ac*- or *bc*-plane.

Here, the phenomena we seek to quantify is that for the two groups of rings angled about the *ac* and *bc* plane, rings above, below, left, and right of a given ring are in *opposite* rotations, whereas rings in the four *corners* surrounding a given ring are in the *same* rotation. Representing + and – as white and black spaces, we seek to mathematically characterize a chessboard pattern. Taking inspiration from the Ising model, we label the configuration of ring *i* as $s_i = +1$ for a positive

rotation and $s_i = -1$ for a negative rotation and develop the following "energetic" (with the analogy to the Ising Model) order parameter to quantify the degree to which ring i is in a chessboard configuration:

$$\sum_{j\ =\ up,down,left,\ right} s_i s_j + \sum_{j\ =\ corners} - s_i s_j$$

The rings above, below, left, and right of a given ring contribute a -1 if the rings are in the *opposite* configuration and the four corners contribute a -1 if the rings are in the *same* configuration. This order parameter is thus a more negative number when the rings are in a checkerboard orientation. We compute this order parameter separately for both the set of rings more aligned with the *ac*-plane and the set of rings more aligned with the *bc*-plane.

The resulting order parameters as a function of chemical potential of Xe (pressure) are shown in Fig S16. As the pressure of Xe increases, one can see that (1) the rings in a line down the *c*-axis align, as entropy reduces to zero, (2) rings in planes parallel to the *ab*-plane adopt a chessboard pattern (the "energetic" order parameter decreases).

A

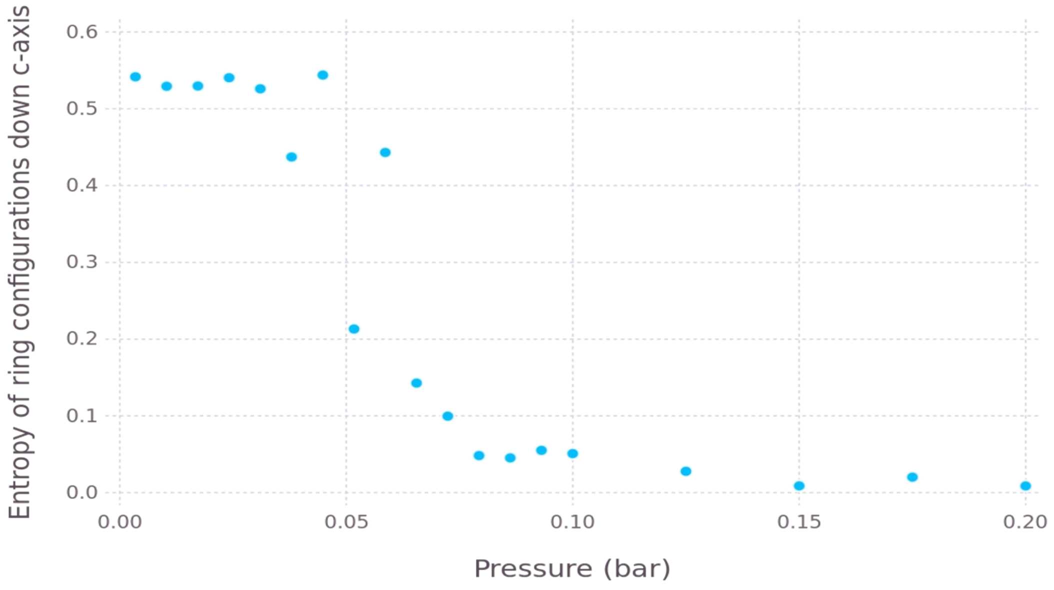

B

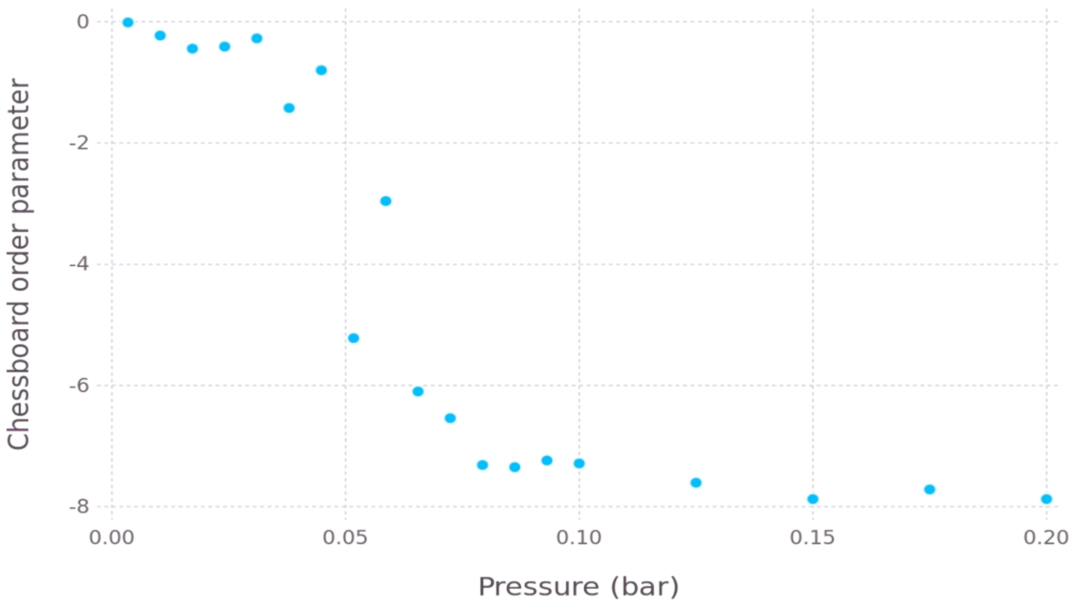

Figure S16. Order parameters describing organization of pyz rings in SIFSIX-3-Ni in our grand-canonical Monte Carlo simulations of Xe adsorption with flipping pyz rings. For perspective, the

structure in Fig S19 corresponds to an entropy of ring configurations down the *c*-axis of zero and chessboard order parameter of -8. (a) Entropy order parameter decreases to zero as the pressure increases. (b) Chessboard order parameter decreases to -8 as the pressure increases.

Note on crystal structures used for molecular simulations

For all computational results, we used the DFT-optimized (see below) crystal structures of Ni- and Fe-SiF6-pyz, but we replaced the pyrazine ring with that from the TraPPE force field for consistency with our molecular models; the center of the pyrazine ring was placed at fractional coordinates [0.0, 0.5, 0.5] and [0.5, 0.0, 0.5]. We provide as supplementary data the DFT-optimized crystal structures.

DFT calculations

All DFT calculations were performed with the Vienna Ab initio Simulation Package (VASP)[7] version 5.3.5, using projector augmented wave method (PAW) potentials[8] generated[9] using PBE[10]. We used a revised version of the van der Waals dispersion-corrected density functional (rev-vdW-DF2)[11] that was implemented in VASP by Klimeš et al.[12] using the algorithm of Román-Pérez and Soler[13]. Hubbard U corrections[14] of 4.0 and 6.4 eV were applied to the localized d electrons of Fe and Ni, respectively, with the U values chosen to reproduce experimental oxidation energies[15] this procedure has been shown to lead to reasonable lattice constants and bond lengths in other MOFs[16].

Periodic DFT optimizations of both MOF structures were done beginning with the 28-atom tetragonal primitive unit cell of the SIFSIX-3-Ni structure obtained by PXRD under a helium atmosphere. The primitive unit cell contains two pyrazine rings, each of which is oriented at an angle to the c-axis; by replicating the primitive unit cell in a periodic manner, the cage adopts an IN-IN-OUT-OUT configuration. Integration over the Brillouin zone was carried out over a 4×4×4 Γ-centered Monkhorst-Pack grid. The plane-wave basis set was cutoff at 1,000 eV, and the wavefunction energy convergence criterion was set to 10^{-6} eV. The a and c lattice vectors were varied in increments of 0.01 Å, and at each of these grid points the atomic positions were optimized until all forces were smaller than 0.01 eV $Å^{-1}$. The total crystal energies were fit to a fourth-order polynomial with respect to the lattice vectors to find the optimal lattice vectors[17]. For both MOF structures investigated, the high-spin state was found to be the ground state and was used in the Monte Carlo simulations. The resulting lattice parameters and bond lengths show good agreement with experiment (Fig S17). The nuclear coordinates of the relaxed structures are included in this work in the format of VASP POSCAR files.

To calculate energies of the different pyrazine ring configurations with and without Xe, the optimized primitive unit cell for each metal was replicated to form a 2×2×2 super-cell, and each cage's pyrazine rings were then flipped to the OUT-OUT-OUT-OUT configuration on one side of the cage and the IN-IN-IN-IN configuration on the other side. One of the groups of four pyrazine rings was then changed from OUT-OUT-OUT-OUT to one of four configurations that

we investigated: OUT-OUT-OUT-OUT (no change required), IN-OUT-OUT-OUT, IN-OUT-IN-OUT, and IN-IN-OUT-OUT. Single point energies were then calculated, from which it was found that for both metals, the IN-OUT-IN-OUT configurations were lowest in energy. Then, eight Xe atoms were placed in the structure, with each atom next to an OUT-OUT-OUT-OUT configuration (or what was originally an OUT-OUT-OUT-OUT configuration) of pyrazine rings at the experimental distance away. The positions of the Xe atoms were then allowed to relax until all forces were smaller than 0.01 eV $Å^{-1}$ while the MOF atoms were kept rigid, from which it was found that for both metals, the OUT-OUT-OUT-OUT configurations were lowest in energy. Integration over the Brillouin zone was carried out over a 2×2×2 Γ-centered Monkhorst-Pack grid. The plane-wave basis set was cutoff at 600 eV, and the wavefunction energy convergence criterion was set to 10^{-6} eV. The nuclear coordinates of the relaxed Xe-containing structures are included in this work in the format of VASP POSCAR files.

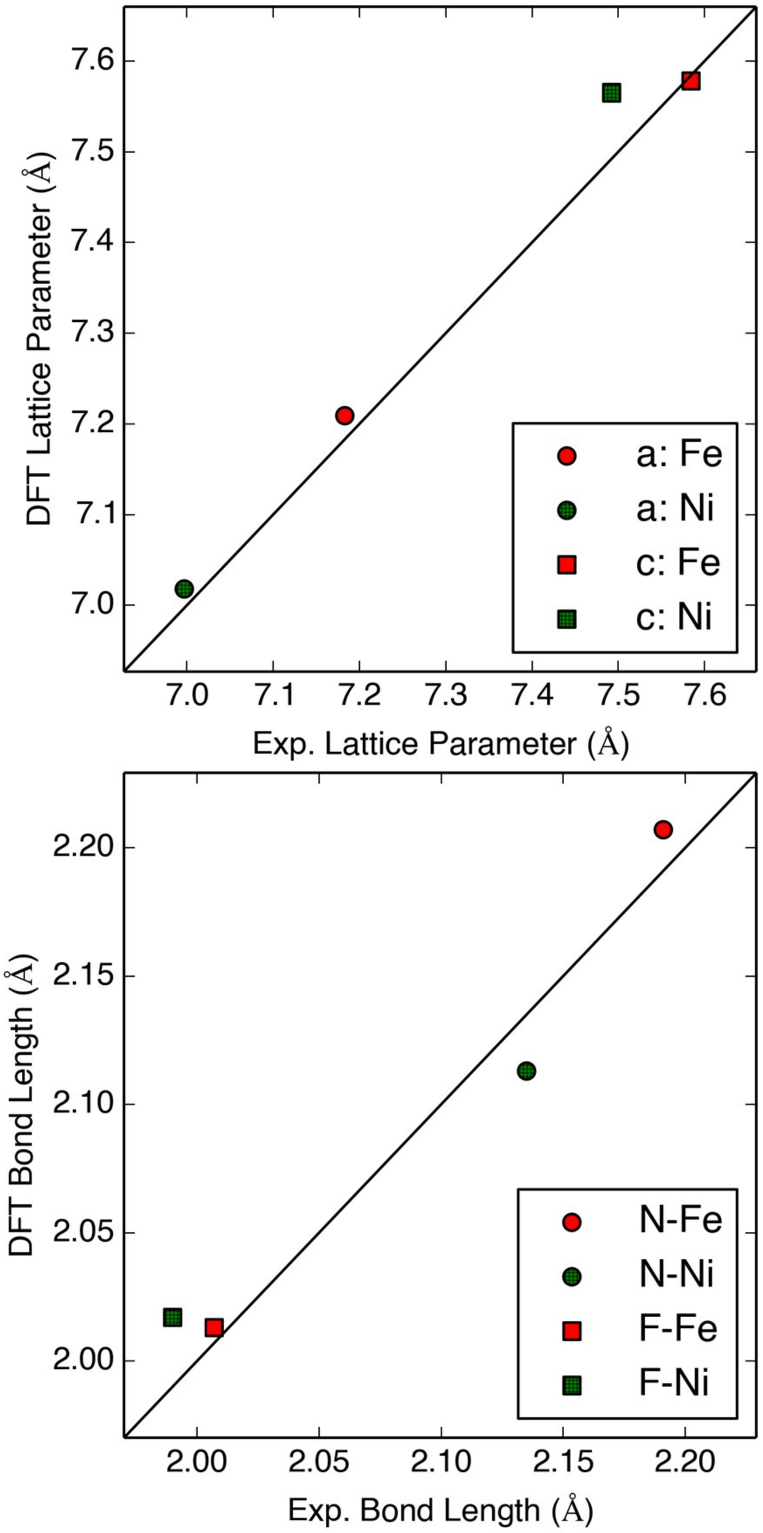

Figure S17. (top) Lattice parameters of the experimental and DFT-relaxed unit cells. (bottom) Nitrogen-metal and fluorine-metal bond lengths of the experimental and DFT-relaxed unit cells.

A

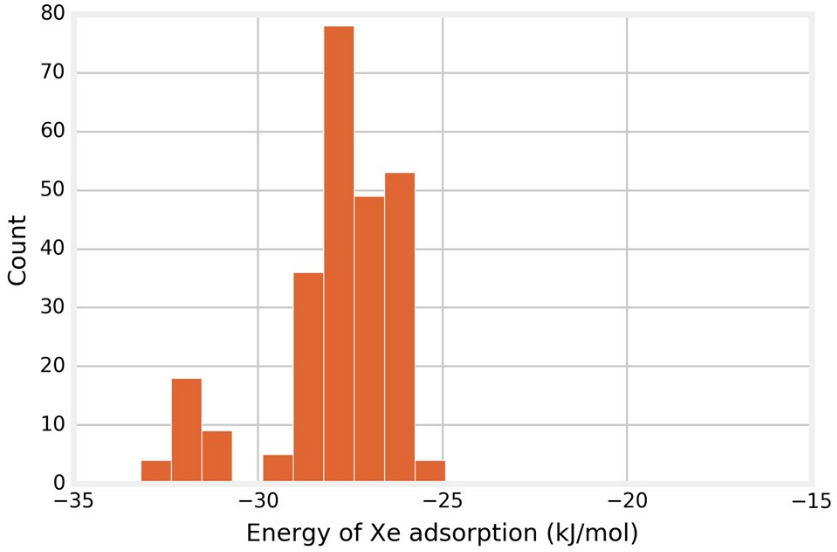

B

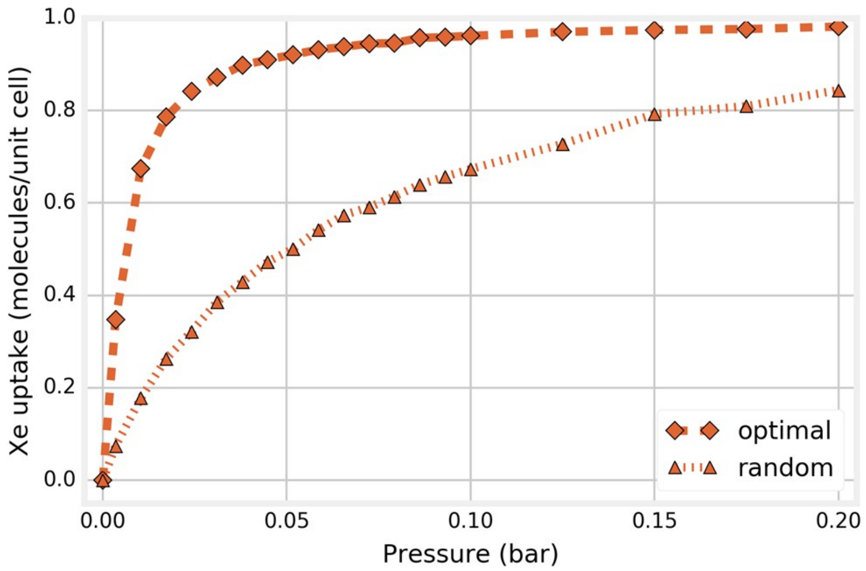

Figure S18. Fe-SiF6-pyz. (A) Distribution of Xe adsorption energies among the 2^8 configurations of pyz ring rotations in a cage. Note that, compared to Ni, the distribution spans a smaller interval of energies and that there are only two clusters. (B) Two simulated rigid-host Xe isotherms. In one host, the ring rotations are chosen at random. In the other host, the rings are

aligned to achieve the optimal Xe energy of adsorption in each cage (OUT-OUT-OUT-OUT on one side of the cage and IN-IN-IN-IN on the other).

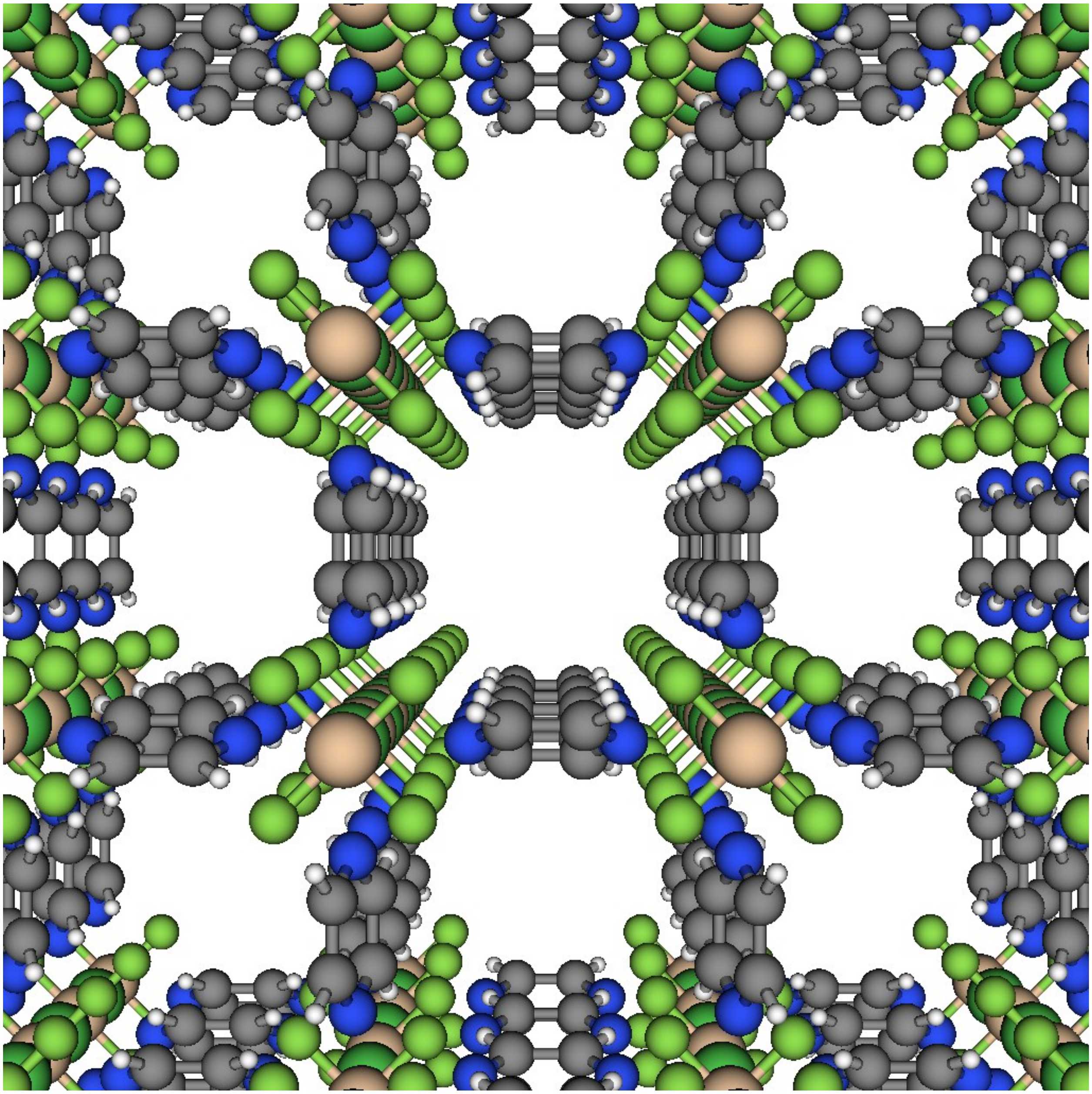

Figure S19. Configuration of rings that yields optimal Xe energy of adsorption in SIFSIX-3-Ni. This view is down the *c*-axis. The pyrazine rings adopt rotational configurations so that every cage achieves the optimal Xe energy of adsorption in SIFSIX-3-Ni seen in Fig 5a. To achieve

this, (1) all rings in a given line down the *c*-axis are aligned (2) the rings in the *b*-*c* plane and *a*-*c* plane form a chessboard pattern in their alignment.

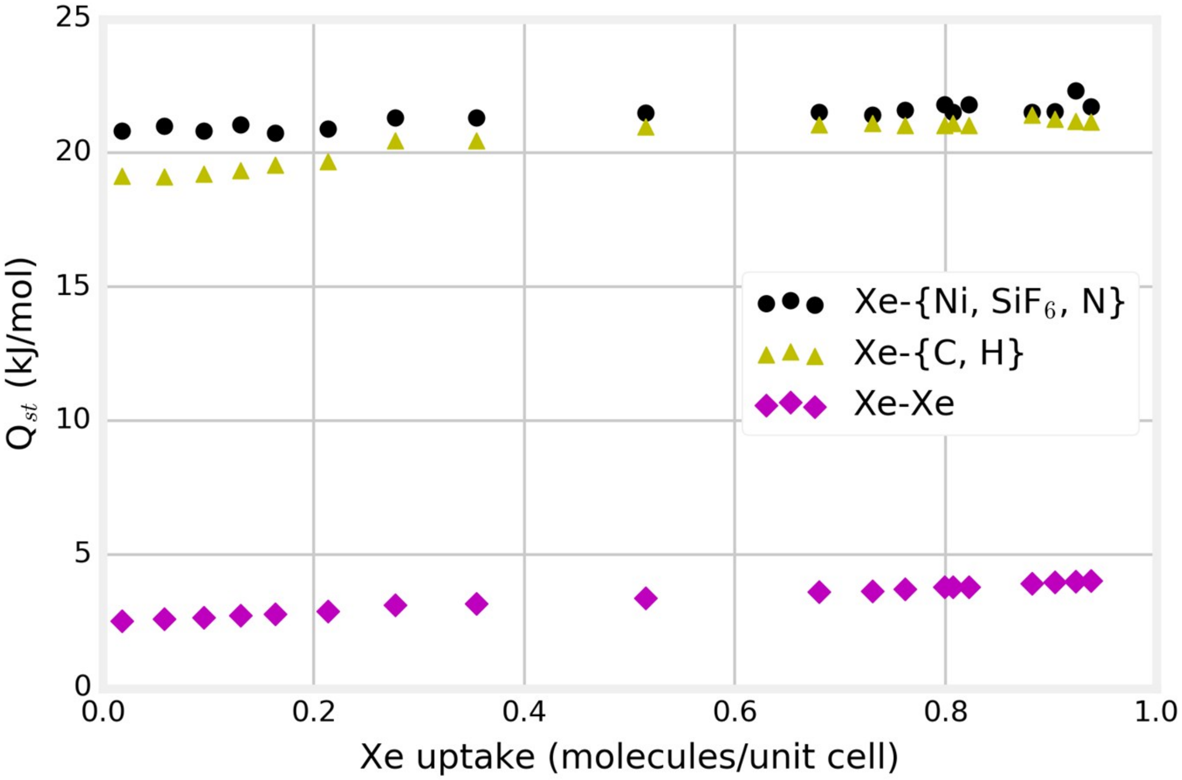

Figure S20. Isosteric heat of adsorption during simulated Xe adsorption SIFSIX-3-Ni. The heat of adsorption is partitioned into Xe-(Ni, SiF6,N), Xe-(C,H) and Xe-Xe interactions. We see that the isosteric heat of adsorption increases with Xe uptake as the rings gradually align to achieve a greater guest-host interaction.

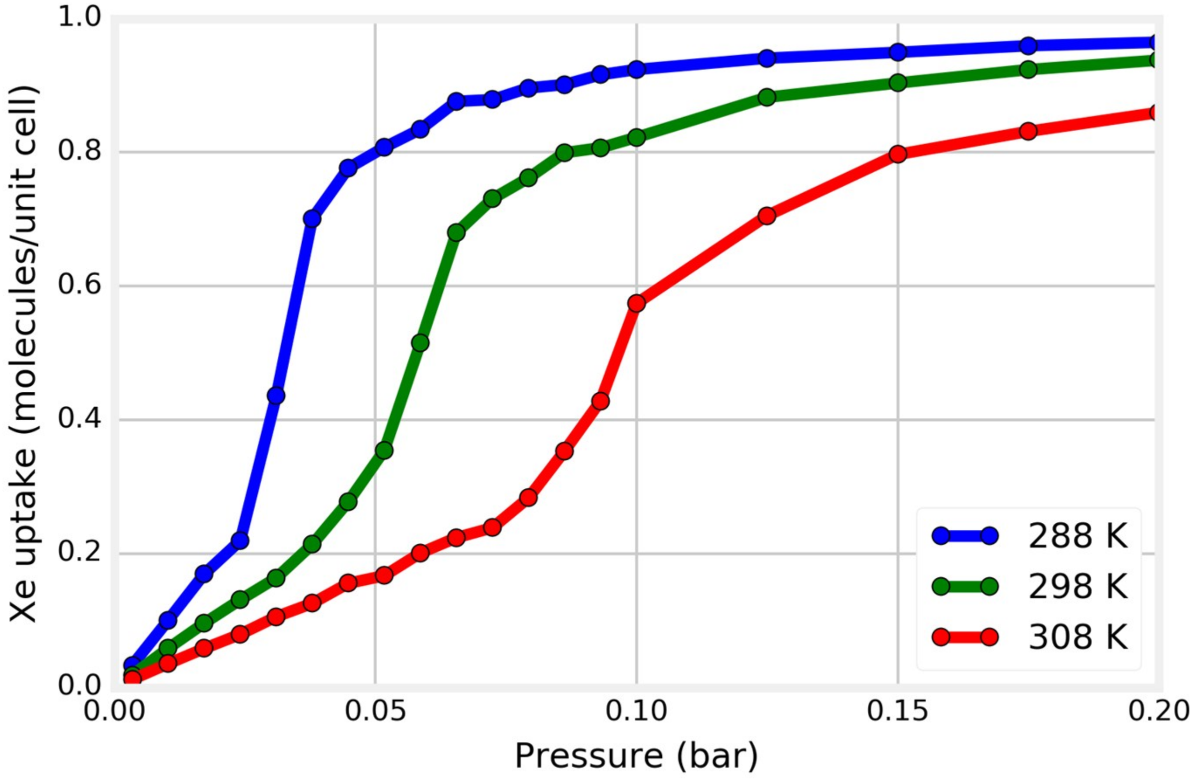

Figure S21. Simulated isotherms in SIFSIX-3-Ni with freely flipping rings at three different temperatures (288 K, 298 K, 313 K). The inflection broadens and shifts to higher pressures as temperature increases, in qualitative agreement with the experiments (see main text).

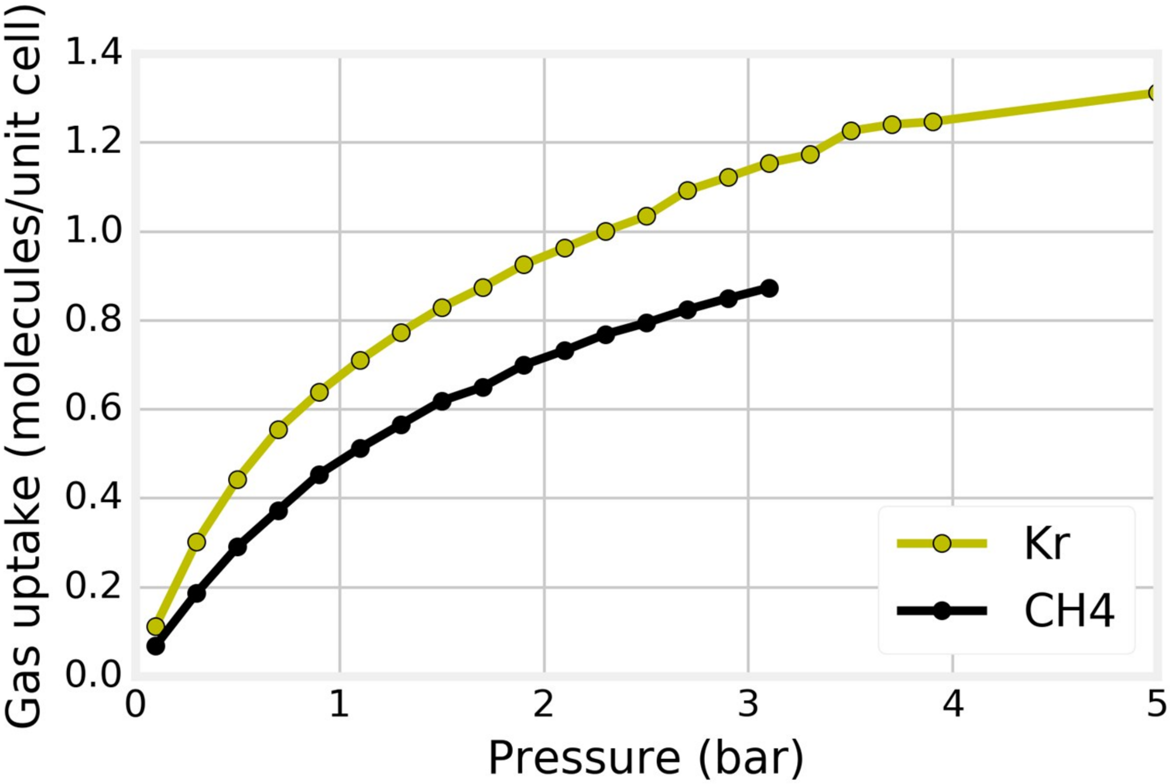

Figure S22. Simulated adsorption isotherms of Kr (yellow) and methane (black) in SIFSIX-3-Ni with flipping pyrazine rings (298 K). Note the absence of an inflection point, in contrast to Xe.

In situ PXRD

The *in stu* PXRD experiment was performed at Beamline 17-BM of Advanced Photon Source using monochromatic radiation (λ = 0.72768 Å). Powder samples of SIFSIX-3-Ni were loaded into 1.1 mm diameter amorphous silica capillaries which were then mounted to an environmetal cell setup.[8] The samples were activated at 80 ˚C under 1 atm He environment until no observable change of the XRD profiles, which took less than 30 minutes. The samples were then cooled to room temperature, at which He was replaced with Xe flow through a multi-channel mass flow controller. For the measurement under vacuum, the same environmental cell setup was connected to a mechanical pump that brought pressure to 0.1 Pascal at room temperature. Rietveld refinement was carried out with program GSAS.[9] In the refinement, restraints were applied to C-C, C-N, C-H bond lengths and C-N-C, N-C-H bond angles in the pyrazine group considering that the bond lengths in the pyrazine ring are approximately rigid and insignificantly influenced by other parts of the structure. Table S2 lists the applied restraints in the three refinements. The displacement factor (Uiso) for H was fixed to be 1.2 times of that for C as a reasonable approximation, which is commonly used in the single crystal structure solutions. Refinement fittings are plotted in Figure S23 to S25. The related crystallographic information files (CIFs) are attached as separate supporting documents. It was found the pyrazine ring faces in the structure are not parallel with the ac/bc plane of the unit cell, but intercept the planes at an angle of ~16°. This angle is derived from refined fractional coordinates of the C atom and the cell lattice constants using this equation:

$$\tan(\varphi) = \frac{b|Y_c - Y_N|}{c|Z_c - Z_N|}$$

in which φ is the tilting angle of the pyrazine ring from the ac plane, Y_C, Z_C, Y_N and Z_N are the Y and Z fractional coordinates of the C atom and the N atom of the pyrazine, and b and c are the unit cell lattice constants. An analogoues equation holds for the rings whose faces are more tangential to the bc plane. The N atom locates at a special position that Y_N and Z_N are both 0.5. Table S1 summarizes the refinement statistics and the key parameters used to calculate the tilting angles. Note that similar tilting of the pyrazine ring was also found in SIFSIX-3-Fe, the structure of which was determined from single crystal diffraction data in this study. This slanting of the

pyrazine rings with respect to the crystallographic planes observed with XRD is consistent with the minimum energy rotational configuration of the pyrazine rings in our DFT calculations.

Table S1 Refinement statistics and parameters of SIFSIX-3-Ni and SIFSIX-3-Fe.

	R (%)	a/b (Å)	c (Å)	Y_C	Z_C	Φ (°)
SIFSIX-3-Ni (He)	3.5	6.9973(2)	7.4918(3)	0.4562(10)	0.6496(3)	15.3
SIFSIX-3-Ni (Xe)	4.1	7.0187(4)	7.5098(5)	0.4541(14)	0.6479(4)	16.2
SIFSIX-3-Ni (Vacuum)	3.1	7.0012(2)	7.4979(2)	0.4482(7)	0.6465(3)	18.3
Fe-SiF6-pyz*	5.9	7.1831(11)	7.5839(12)	0.4610(14)	0.3558(9)	14.4

* based on single crystal x-ray diffraction data

Table S2 Restraints used in the PXRD refinements of SIFSIX-3-Ni and the refined values

	Restaints		Refined results		
	Value	Sigma	He-loaded	Xe-loaded	Vacuum
C-N bond length (Å)	1.339	0.001	1.339	1.339	1.340
C-C bond length	1.393	0.001	1.394	1.392	1.395
C-H bond length	1.080	0.005	1.115	1.105	1.107
C-N-C bond angle (°)	121.85	0.10	120.27	119.39	119.42
N-C-H bond angle	115.95	0.10	116.95	116.92	116.80

Note the restraints are not hard numbers to bind to, but another guage when fitting is evaluated. Therefore, the refined values may deviate from set values upon convergence. Also note for the SIFSIX-3-Fe structure solved from single crystal data, no bond length or angle restraints were applied.

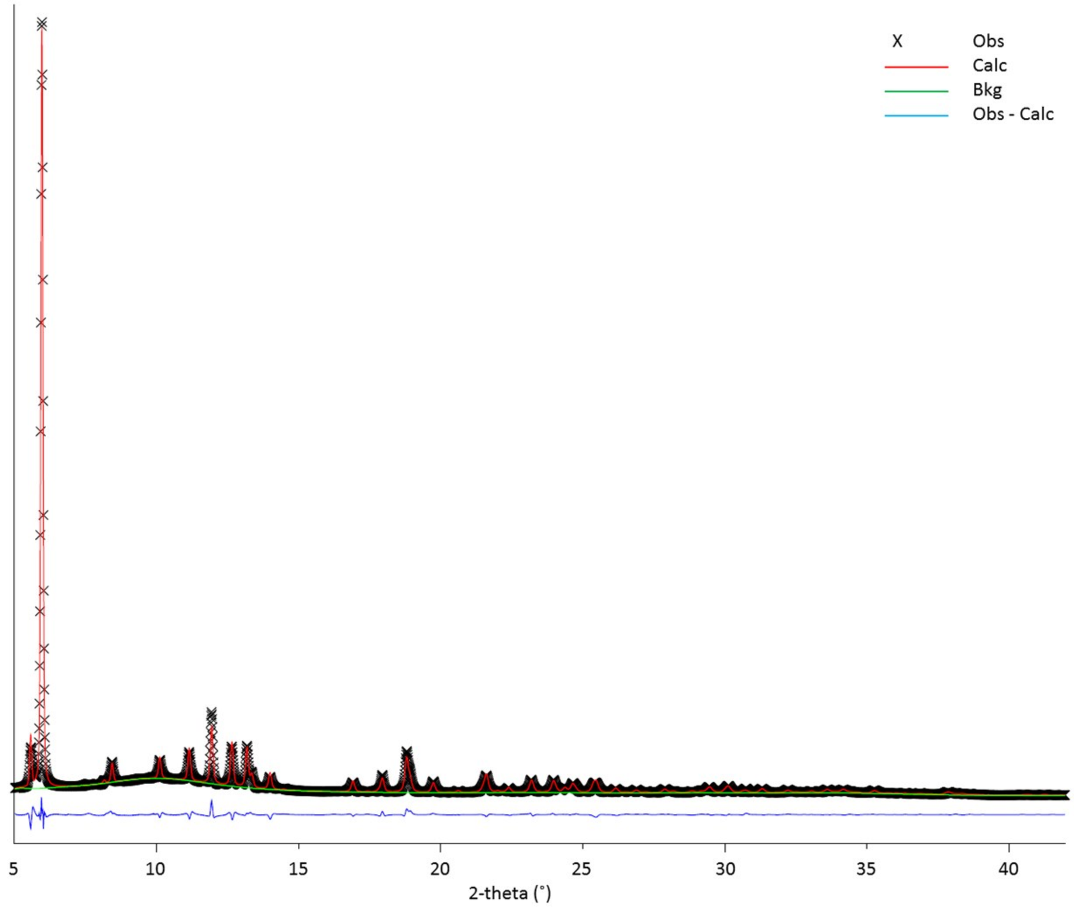

Figure S23. Refinement fitting plot of the in situ PXRD data on He-loaded SIFSIX-3-Ni.

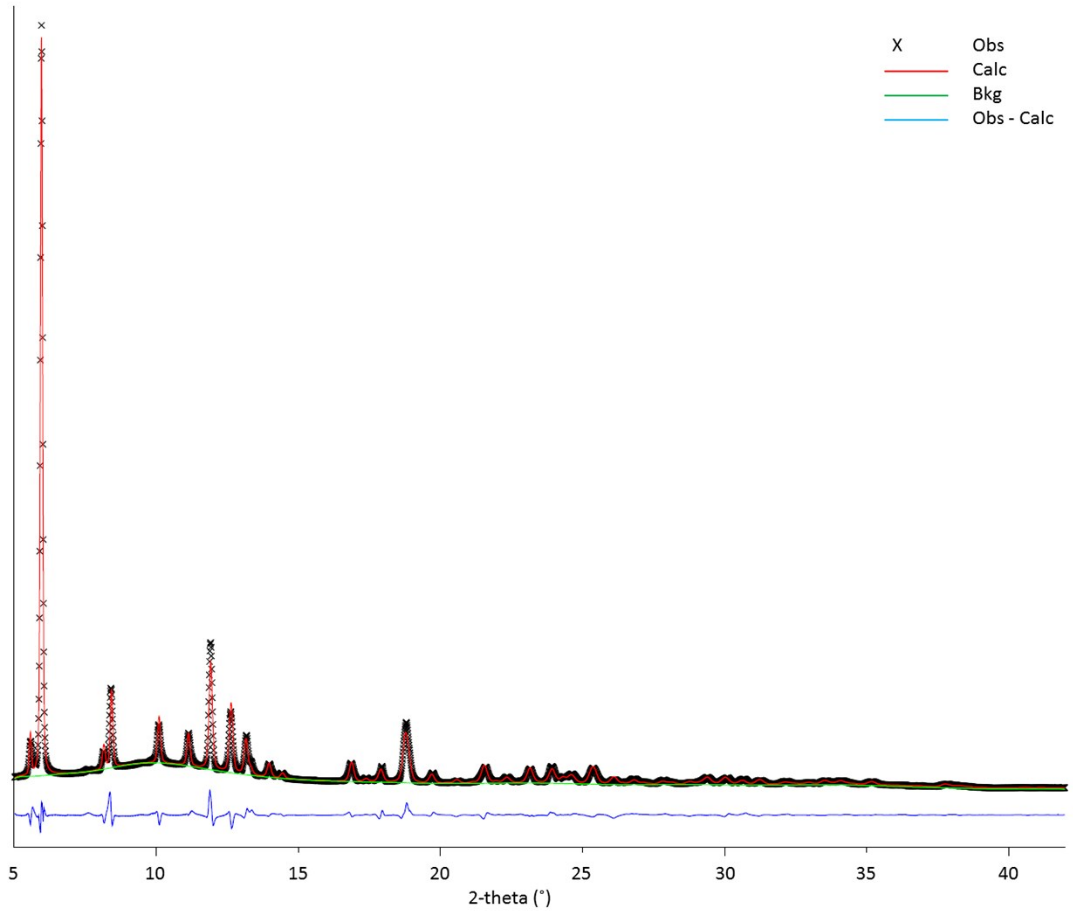

Figure S24. Refinement fitting plot of the in situ PXRD data on Xe-loaded SIFSIX-3-Ni.

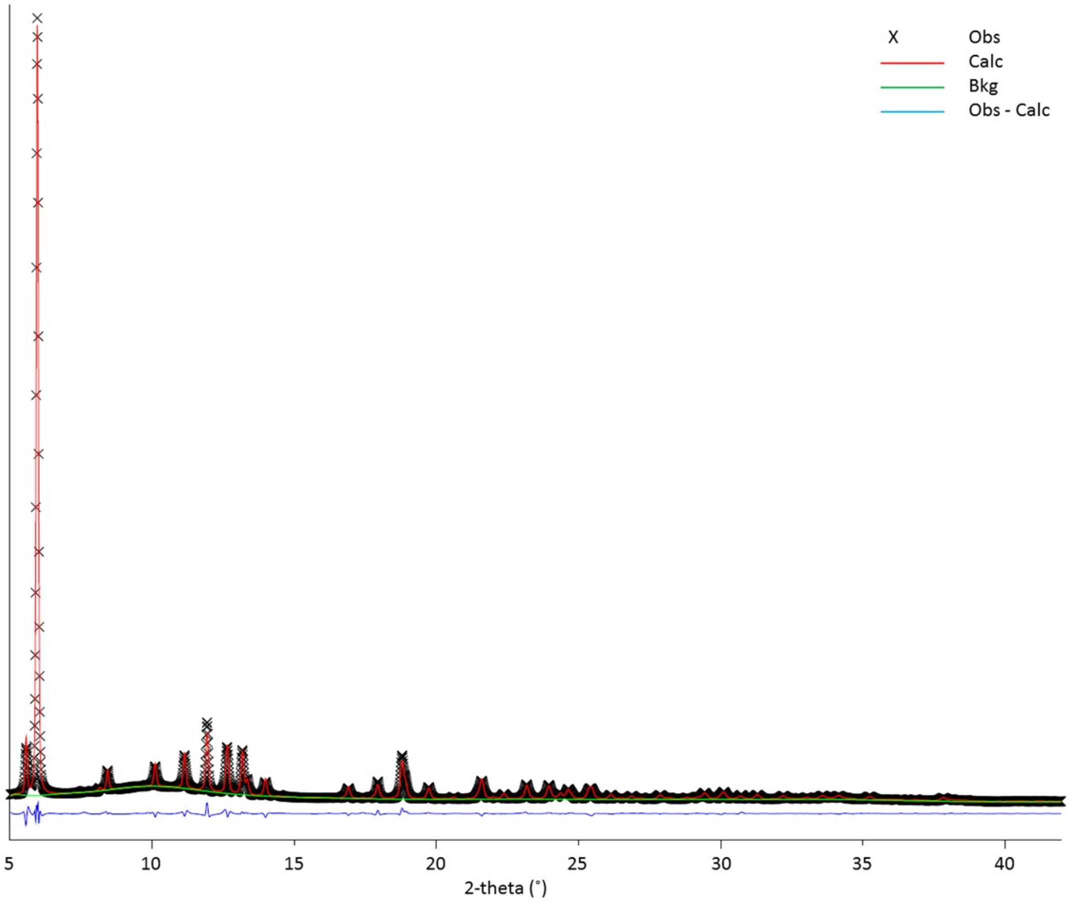

Figure S25. Refinement fitting plot of the in situ PXRD data on SIFSIX-3-Ni under vacuum.

Additional References:

(1) Hirschfelder, J. O.; Curtiss, C. F.; Bird, R. B. *Molecular Theory of Gases and Liquids*; Wiley, New York: New York

1965.

(2) Rai, N.; Siepmann, J. I. *The Journal of Physical Chemistry B* **2007**, *111*, 10790.

(3) Mayo, S. L.; Olafson, B. D.; Goddard, W. A. *The Journal of Physical Chemistry* **1990**, *94*, 8897.

(4) Rappe, A. K.; Casewit, C. J.; Colwell, K. S.; Goddard, W. A.; Skiff, W. M. *Journal of the American Chemical Society* **1992**, *114*, 10024.

(5) Frenkel, D.; Smit, B. *Understanding molecular simulation: from algorithms to applications* Academic press, 2001; Vol. 1.

(6) Coudert, F.-X.; Boutin, A.; Jeffroy, M.; Mellot-Draznieks, C.; Fuchs, A. H. *ChemPhysChem* **2011**, *12*, 247.

(7) Kresse G.; Furthmüller, J. *Phys. Rev. B*, **1996**, *54*, 11169–11186.

(8) Blöchl, P. E. *Phys. Rev. B*, **1994**, *50*, 17953–17979.

(9) Kresse G.; Joubert, D. *Phys. Rev. B*, **1999**, *59*, 1758–1775.

(10) Perdew, J. P.; Burke K.; Ernzerhof, M. *Phys. Rev. Lett.*, **1996**, *77*, 3865–3868.

(11) Hamada, I. *Phys. Rev. B*, **2014**, *89*, 121103.

(12) Klimeš, J.; Bowler, D. R.; Michaelides, A. *Phys. Rev. B*, **2011**, *83*, 195131.

(13) Román-Pérez, G.; Soler, J. M. *Phys. Rev. Lett.*, **2009**, *103*, 096102.

(14) Dudarev, S. L.; Botton, G. A.; Savrasov, S. Y.; Humphreys, C. J.; Sutton, A. P. *Phys. Rev. B*, **1998**, *57*, 1505–1509.

(15) Wang, L.; Maxisch, T.; Ceder, G. *Phys. Rev. B*, **2006**, *73*, 195107.

(16) Lee, K.; Howe, J. D.; Lin, L.-C.; Smit, B.; Neaton, J. B. *Chem. Mater.*, **2015**, *27*, 668–678.

(17) Ziambaras E.; Schröder, E. *Phys. Rev. B*, **2003**, *68*, 064112.
